# Differential Etv2 threshold requirement for endothelial and erythropoietic development

**DOI:** 10.1016/j.celrep.2022.110881

**Published:** 2022-05-31

**Authors:** Tanvi Sinha, Kelly Lammerts van Bueren, Diane E. Dickel, Ivana Zlatanova, Reuben Thomas, Carlos O. Lizama, Shan-Mei Xu, Ann C. Zovein, Kohta Ikegami, Ivan P. Moskowitz, Katherine S. Pollard, Len A. Pennacchio, Brian L. Black

**Affiliations:** 1 Cardiovascular Research Institute, University of California, San Francisco, San Francisco, CA 94143, USA; 2 Environmental Genomics and Systems Biology Division, Lawrence Berkeley National Laboratory, Berkeley, CA 94720, USA; 3 U.S. Department of Energy Joint Genome Institute, 1 Cyclotron Road, Berkeley, CA 94720, USA; 4 Comparative Biochemistry Program, University of California, Berkeley, Berkeley, CA 94720, USA; 5 Gladstone Institutes, San Francisco, San Francisco, CA 94158, USA; 6 Division of Molecular and Cardiovascular Biology, Cincinnati Children’s Hospital Medical Center, Cincinnati, OH 45229, USA; 7 Department of Pediatrics, University of Cincinnati, Cincinnati, OH 45229, USA; 8 Departments of Pediatrics, Pathology, and Human Genetics, University of Chicago, Chicago, IL 60637, USA; 9 Department of Epidemiology and Biostatistics, University of California, San Francisco, San Francisco, CA 94143, USA; 10 Chan Zuckerberg Biohub, San Francisco, San Francisco, CA 94158, USA; 11 Department of Biochemistry and Biophysics, University of California, San Francisco, San Francisco, CA 94143, USA; 12 Lead contact

## Abstract

Endothelial and erythropoietic lineages arise from a common developmental progenitor. Etv2 is a master transcriptional regulator required for the development of both lineages. However, the mechanisms through which Etv2 initiates the gene-regulatory networks (GRNs) for endothelial and erythropoietic specification and how the two GRNs diverge downstream of Etv2 remain incompletely understood. Here, by analyzing a hypomorphic *Etv2* mutant, we demonstrate different threshold requirements for initiation of the downstream GRNs for endothelial and erythropoietic development. We show that Etv2 functions directly in a coherent feedforward transcriptional network for vascular endothelial development, and a low level of *Etv2* expression is sufficient to induce and sustain the endothelial GRN. In contrast, Etv2 induces the erythropoietic GRN indirectly via activation of Tal1, which requires a significantly higher threshold of *Etv2* to initiate and sustain erythropoietic development. These results provide important mechanistic insight into the divergence of the endothelial and erythropoietic lineages.

## In brief

Sinha et al. demonstrate that the master regulatory transcription factor Etv2 directly regulates the mouse embryonic endothelial gene-regulatory network (GRN). In contrast, Etv2 regulates the erythropoietic GRN indirectly via the bHLH transcription factor Tal1, making erythropoiesis more sensitive than vasculogenesis to reduced Etv2 gene dosage.

## Graphical Abstract

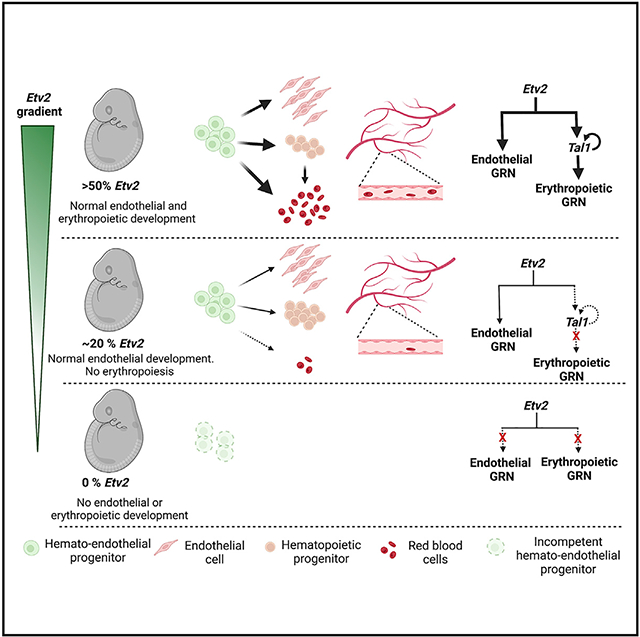

## INTRODUCTION

Hematopoiesis is the process by which all blood cells, including erythrocytes, myeloid cells, and lymphoid cells, are formed (Dzierzak and Speck, 2008). During mouse embryogenesis, blood development can be divided into two broadly defined waves, primitive and definitive hematopoiesis (Baron, 2013; Dzierzak and Speck, 2008). Primitive hematopoiesis begins at around embryonic day (E)7.0 in the extraembryonic yolk sac and produces red blood cells (erythropoiesis) and macrophage and megakaryocyte progenitors (Baron, 2013; Palis, 2014). The early wave of erythropoiesis is essential for embryonic survival, and loss of the primitive erythropoietic population is associated with early embryonic lethality (Baron, 2013). The early wave of primitive erythropoiesis is succeeded by definitive hematopoiesis, marked by the appearance of the hemogenic endothelium and definitive hematopoietic progenitor cells in the yolk sac and in the aorta-gonad-mesonephros (AGM) region of the embryo (Gritz and Hirschi, 2016). Indeed, from the earliest stages of primitive hematopoiesis, there is an intimate association of blood and endothelial cell development. Both cell types initially originate from “blood islands” in the yolk sac, and numerous studies have established that the development of blood and endothelial cells are coupled during both primitive and early definitive hematopoiesis (Baron, 2013).

Given the close association of blood and endothelial lineages, it is not surprising that the transcription factors controlling their development are overlapping, especially in the earliest steps of their gene-regulatory networks (GRNs) (Lee et al., 2008; Menegatti et al., 2019; Robb et al., 1995; Shivdasani et al., 1995; Visvader et al., 1998). The earliest described master regulator of hemato-endothelial development in the mouse is the Ets variant 2 (Etv2) transcription factor (Garry, 2016; Lammerts van Bueren and Black, 2012; Sumanas and Choi, 2016). Etv2 is expressed transiently in hemato-endothelial progenitors in the mesoderm of the early mouse embryo between E7.0 and E9.0 (Ferdous et al., 2009; Lee et al., 2008). Loss of function for *Etv2* results in early lethality before mid-gestation due to a complete absence of all hemato-endothelial lineages (Ferdous et al., 2009; Lee et al., 2008). Similarly, zebrafish *etv2* mutants display defects in hemato-vascular development (Pham et al., 2007; Sumanas et al., 2008; Sumanas and Lin, 2006). The importance of Etv2 in the hematopoietic and endothelial lineages is further underscored by its role in converting fibroblasts and other cell types to endothelial or hematopoietic fates, particularly in combination with Forkhead transcription factors (De Val et al., 2008; Ginsberg et al., 2012, 2015; Lee et al., 2008).

Etv2 functions at the top of the GRNs for both endothelial cell and erythropoietic development and activates multiple important regulators of both networks, including Ets1, Fli1, Tal1 (Scl), and Gata1 (Lammerts van Bueren and Black, 2012; Liu et al., 2015; Sumanas and Choi, 2016). Of these, Tal1 and Gata1 serve as key transcriptional regulators of primitive erythropoietic development in the yolk sac, and the loss of either of these factors in the mouse results in profound anemia and early embryonic demise (Fujiwara et al., 1996; Pevny et al., 1991; Robb et al., 1995; Shivdasani et al., 1995). Concomitantly, the Ets transcription factors Ets1 and Fli1 are important regulators of endothelial development (Bloor et al., 2002; De Val and Black, 2009; Meadows et al., 2011). How the hemato-endothelial transcriptional hierarchy diverges downstream of Etv2 to regulate the GRNs of the two closely related lineages has been challenging to define, due, in part, to the transient expression of Etv2 in early common progenitors and the inability to abolish Etv2 function in the earliest progenitors without affecting both lineages (Ferdous et al., 2009; Kataoka et al., 2013; Koyano-Nakagawa and Garry, 2017; Lee et al., 2008). Similarly, it has been difficult to determine whether the loss of the hematopoietic lineage in *Etv2* mutants is due to the failure of endothelial specification or due to an autonomous role for Etv2 in hematopoietic development. For instance, deletion of *Etv2* in the endothelial lineage using *Tie2*::*Cre* or in the hematopoietic lineage using *Vav1*::*Cre* failed to recapitulate any of the severe vascular or hematopoietic defects observed in *Etv2*-null mouse mutants (Kataoka et al., 2013). In contrast, inactivation of *Etv2* broadly in the early mesoderm using *Mesp1*::*Cre* recapitulated the severe hemato-endothelial defects observed in *Etv2*-null mutants, precluding a specific analysis of only the hematopoietic lineages (Kataoka et al., 2013).

Here, by deleting an upstream enhancer of *Etv2*, we generated a hypomorphic *Etv2* allele with reduced *Etv2* expression. Using various combinations of this hypomorphic allele with *Etv2*-null and wild-type alleles, we generated an allelic series with *Etv2* expression ranging from 100% to 0% of normal. Interestingly, a compound heterozygous combination with ~20% of normal *Etv2* expression resulted in embryonic lethality at E10.5 with severe anemia but with apparently normal vascular development. Mechanistically, we show that this differential dose requirement for Etv2 in erythropoietic versus endothelial development is due to distinct mechanisms of regulation of the endothelial and erythropoietic GRNs downstream of Etv2. Etv2 regulates early endothelial development via activation of a coherent, feedforward transcriptional circuit and corresponding direct binding to a significant fraction of early endothelial gene enhancers. As a result, expression of those genes is relatively resistant to hypomorphic Etv2 dose. In contrast, Etv2 regulates the erythropoietic GRN indirectly via activation of Tal1, which in turn functions in combination with Gata1 to activate early erythropoietic gene enhancers, making the early erythropoietic program inherently unstable and highly sensitive to Etv2 dose. These results have important implications for our understanding of the relationship between endothelial and erythropoietic development *in vivo*.

## RESULTS

### A proximal upstream *Etv2* enhancer regulates *Etv2* expression *in vivo*

A 3.9-kb proximal promoter and enhancer of the *Etv2* gene has been shown previously to direct early endothelial expression *in vivo* (Rasmussen et al., 2011). Consistent with these previous studies, we found that a conserved 3.3-kb region, including the proximal promoter and transcriptional start site, when fused to a β-galactosidase reporter gene was sufficient to direct expression to endothelial progenitors in mouse embryos in a manner nearly identical to the expression of the endogenous *Etv2* gene (Rasmussen et al., 2011; Figure S1). We next used VISTA (Dubchak and Ryaboy, 2006) to identify smaller evolutionarily conserved regions and used that conservation as the basis for dividing the previously described *Etv2* enhancer-promoter element into smaller fragments encompassing proximal and distal regions (Figure S1A). The proximal 804-bp region failed to direct β-galactosidase expression in a reproducible manner or in a pattern consistent with endogenous *Etv2* expression in mouse embryos at E8.5 (data not shown). In contrast, a 1.4-kb fragment encompassing an upstream region of evolutionary conservation (Figure S1A) fused to a minimal *hsp68* promoter and a *lacZ* reporter gene (*Etv2enh*::*lacZ*) efficiently directed β-galactosidase reporter expression in vascular endothelial and hematopoietic progenitor regions of E8.5 transgenic mouse embryos, recapitulating endogenous *Etv2* expression (Figures S1H and S1I).

**Figure 1.**
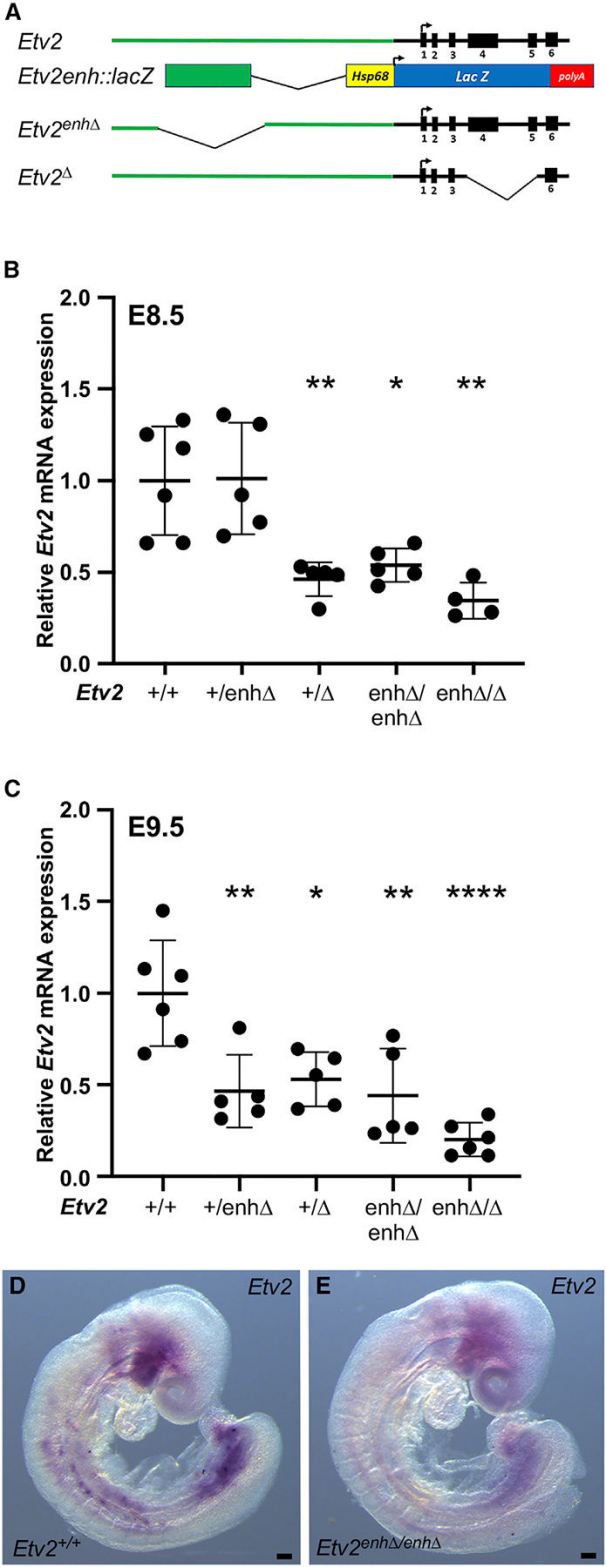
A 1.4-kb upstream enhancer is required for normal levels of *Etv2* expression (A) Schematic representations, in descending order, of the *Etv2* gene locus, *Etv2* enhancer transgene, *Etv2* enhancer mutant (*Etv2*^*enhΔ*^), and *Etv2*-null (Etv2^Δ^) alleles. (B and C) qPCR quantification of endogenous *Etv2* mRNA expression at E8.5 (B)and E9.5 (C) showing *Etv2* expression in wild-type (+/+), *Etv2*^*+/enhΔ*^, *Etv2*^*+/Δ*^, *Etv2*^*enhΔ/enhΔ*^, and *Etv2*^*enhΔ/Δ*^ embryos. Significantly reduced expression compared with wild-type embryos is indicated (*p < 0.05, **p < 0.01, ***p < 0.001, ****p < 0.0001 by one-way ANOVA, followed by Bonferroni’s multiple comparison test). The number of biological samples analyzed for each genotype at each stage is indicated by individual data points on the graphs. Data are presented as mean ± SD. (D) *Etv2 in situ* hybridization in E9.0 embryos (n = 3 embryos/genotype) shows a generalized reduction in *Etv2* expression in *Etv2*^*enhΔ/enhΔ*^ embryos. Scale bars, 100 μm.

The location of the enhancer in the proximal upstream region of the *Etv2* locus and the concordance of enhancer activity with endogenous *Etv2* expression (Figures S1H and S1I) strongly suggest that the 1.4-kb upstream enhancer is a *bona fide Etv2* enhancer and regulates *Etv2* expression in early hemato-endothelial progenitors *in vivo*. We reasoned that deletion of the *Etv2* enhancer might reveal additional insight into the regulation and function of *Etv2 in vivo*. Therefore, we used CRISPR-Cas9 genome editing to delete the upstream enhancer from the genome, creating *Etv2*^*enhΔ*^ mutant mice (Figure 1A). *Etv2*^*+/enhΔ*^ mice were intercrossed, and *Etv2* expression was assessed by quantitative reverse transcriptase (RT)-PCR (qPCR) in wild-type, *Etv2*^*+/enhΔ*^, and *Etv2*^*enhΔ/enhΔ*^ embryos at E8.5 and E9.5 (Figures 1B and 1C). *Etv2* expression in *Etv2*^*enhΔ/enhΔ*^ embryos was significantly reduced at both time points to approximately 40% of the level of expression observed in wild-type embryos (Figures 1B and 1C). Additionally, *in situ* hybridization revealed a general reduction in expression of *Etv2* transcripts throughout the *Etv2* expression domain in *Etv2*^*enhΔ/enhΔ*^ embryos (Figures 1D and 1E). These results indicate that the 1.4-kb upstream enhancer identified here is a *bona fide* enhancer of *Etv2*.

Interestingly, *Etv2*^*enhΔ/enhΔ*^ mice were born at predicted Mendelian frequency, they appeared phenotypically normal at weaning, and they were fertile as adults with no evident phenotype (18 *Etv2*^*+/+*^, 34 *Etv2*^*+/enhΔ*^, 19 *Etv2*^*enhΔ/enhΔ*^; total = 71; χ^2^ = 0.155; p = 0.9255). These results suggest that ~40% of wild-type *Etv2* expression is sufficient for normal development and postnatal life. In contrast, crossing the *Etv2*^*enhΔ*^ allele onto an *Etv2*-null (*Etv2*^*Δ*^) background (see schematic of alleles in Figure 1A) to generate compound heterozygous *Etv2*^*enhΔ/Δ*^ embryos resulted in a more profound reduction in *Etv2* expression at E8.5 and E9.5 (Figures 1B and 1C). Indeed, by E9.5, *Etv2* expression in *Etv2*^*enhΔ/Δ*^ compound heterozygotes was reduced to approximately 20% of the expression observed in wild-type embryos (Figure 1C), and significantly, no *Etv2*^*enhΔ/Δ*^ mice were recovered at weaning (25 *Etv2*^*+/+*^, 30 *Etv2*^*+/enhΔ*^, 25 *Etv2*^*+/Δ*^, 0 *Etv2*^*enhΔ/Δ*^; total = 80; χ^2^ = 27.5; p < 0.0001). These results indicate that 20% of normal *Etv2* expression, as observed in *Etv2*^*enhΔ/Δ*^ mice, is not compatible with viability.

### *Etv2*^*enhΔ/Δ*^ hypomorphic mutants have apparently normal vascular development

**Figure 2.**
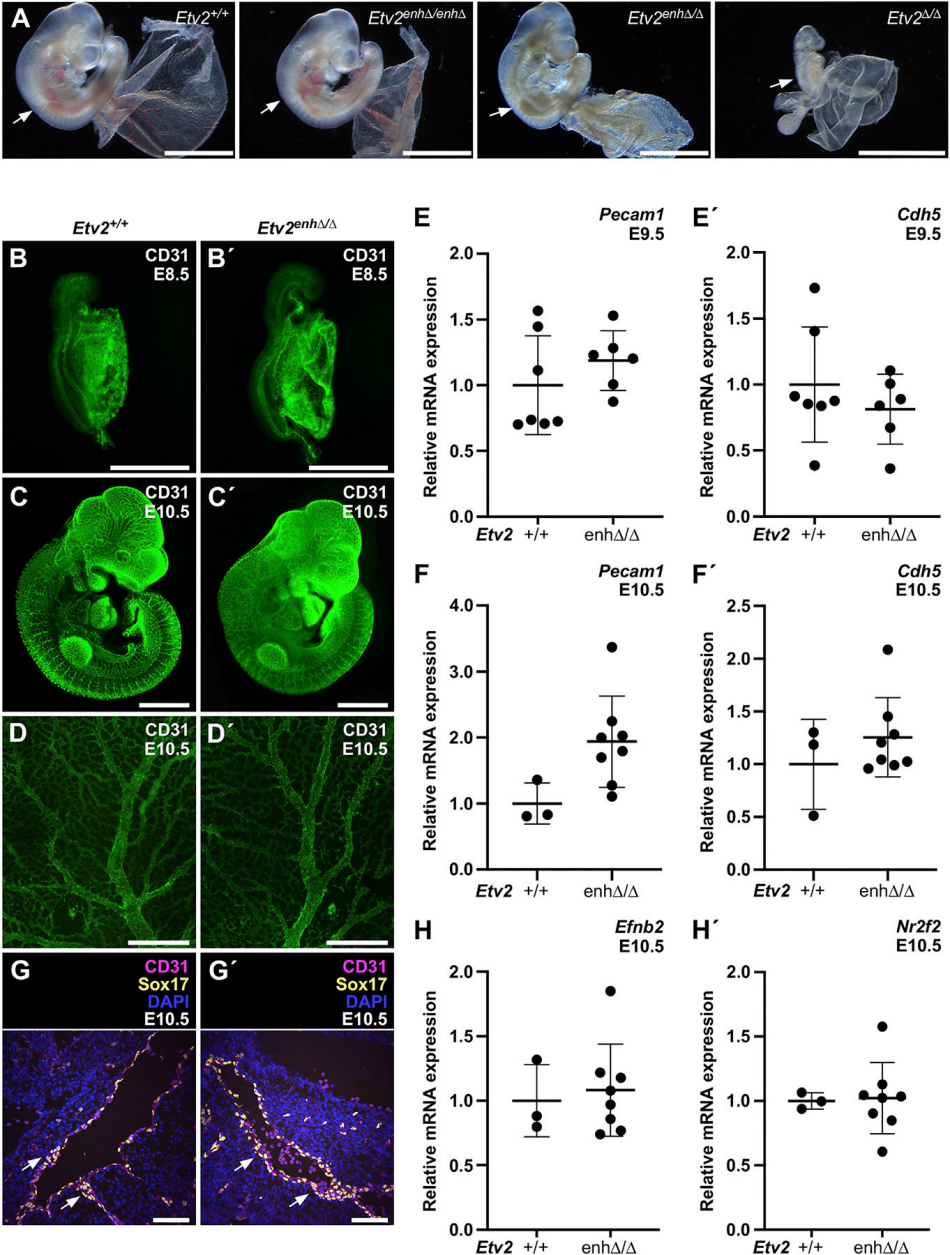
*Etv2*^*enhΔ/Δ*^ compound heterozygotes exhibit apparently normal vascular endothelial development (A) Whole-mount images of *Etv2*^*+/+*^, *Etv2*^*enhΔ/enhΔ*^, *Etv2*^*enhΔ/Δ*^, and *Etv2*^*Δ/Δ*^ embryos and yolk sacs at E10. White arrows point to apparent blood; note anemia in *Etv2*^*enhΔ/Δ*^ and *Etv2*^*Δ/Δ*^ embryos. (B–D) Whole-mount CD31 immunostaining in *Etv2*^*+/+*^ (B–D) and *Etv2*^*enhΔ/Δ*^ (B′–D′) embryos at E8.5 (B and B′), embryos at E10.5 (C and C′), and yolk sacs at E10.5 (D and D′). Note similar level and pattern of CD31 expression between the two genotypes. (E–F) qPCR analyses for *Pecam1* and *Cdh5* in E9.5 (E and E′) and E10.5 (F and F′) show no significant difference in mRNA expression between *Etv2*^*+/+*^ (E and F) and *Etv2*^*enhΔ/Δ*^ (E′ and F′) embryos by unpaired t test. (G) Section immunostaining of the aorta-gonad-mesonephros region of E10.5 embryos shows similar Sox17 expression and pattern in the aorta of *Etv2*^*+/+*^ (G) and *Etv2*^*enhΔ/Δ*^ (G′) embryos (white arrows). (H) qPCR analyses for the arterial marker *EfnB2* (H) and the venous marker *Nr2f2* (H′) in *Etv2*^*+/+*^ and *Etv2*^*enhΔ/Δ*^ embryos. The number of biological samples used for qPCR analysis for each genotype is indicated by individual data points on the graphs. Data are presented as mean ± SD. Scale bars, A, 2 mm; B–D, 1 mm; G, 100 μm. For A–D and G, n = 3 embryos or yolk sacs/genotype.

To gain insight into the lethality observed in hypomorphic *Etv2*^*enhΔ/Δ*^ compound heterozygous mice, we next determined the embryonic stage when defects were first apparent. No *Etv2*^*enhΔ/Δ*^ animals were recovered from *Etv2*^*+/enhΔ*^ × *Etv2*^*+/Δ*^ crosses at E13.5 (6 *Etv2*^*+/+*^, 7 *Etv2*^*+/enhΔ*^, 9 *Etv2*^*+/Δ*^, 0* *Etv2*^*enhΔ/Δ*^; χ^2^ = 8.182; p < 0.0424; *2 partially resorbed embryos were observed). Earlier, at E11.5, some *Etv2*^*enhΔ/Δ*^ embryos were recovered, although many were beginning to be resorbed by that stage (data not shown). In contrast, *Etv2*^*enhΔ/Δ*^ embryos were recovered at predicted Mendelian frequency at E10 (65 *Etv2*^*+/+*^, 76 *Etv2*^*+/enhΔ*^, 72 *Etv2*^*+/Δ*^, 66 *Etv2*^*enhΔ/Δ*^; total = 279; χ^2^ = 1.158, p = 0.7632). Additionally, at E10, *Etv2*^*enhΔ/Δ*^ embryos appeared morphologically normal, had a beating heart, and had no overt defects in the development of the cardiovascular system (Figure 2A), despite only 20% of normal *Etv2* expression (Figure 1C). Expression of the canonical endothelial marker CD31 (PECAM-1) in *Etv2*^*enhΔ/Δ*^ embryos was indistinguishable from expression in wild-type embryos at E8.5 and E10.5 (Figures 2B-2D). Likewise, *Etv2*^*enhΔ/Δ*^ embryos showed a well-remodeled vascular network in the embryo and yolk sac, similar to those of wild-type embryos (Figures 2C and 2D). No significant down-regulation in the expression *Pecam1* or *Cdh5* transcripts in *Etv2*^*enhΔ/Δ*^ embryos was observed by qPCR at either E9.5 or E10.5 (Figures 2E and 2F). The normal expression of endothelial markers and the lack of evident cardiovascular defects in *Etv2*^*enhΔ/Δ*^ hypomorphic embryos was in marked contrast to *Etv2*^*Δ/Δ*^ complete-null embryos, which have a complete loss of *Etv2* expression, never express detectable levels of CD31, and exhibit severe defects in cardiac and vascular development prior to E9.0 (Figure 2A; Ferdous et al., 2009; Lee et al., 2008).

We also examined the expression of markers of arterial and venous development in *Etv2*^*enhΔ/Δ*^ mutant embryos. Co-immunostaining of sagittal sections of E10.5 embryos for expression of CD31 and the early arterial marker Sox17 showed that the arterial endothelium of the dorsal aorta was apparently normally specified in *Etv2*^*enhΔ/Δ*^ embryos, at least with respect to expression of Sox17 (Figure 2G). Likewise, no significant differences in the expression levels of either the arterial marker gene *Efnb2* or the venous marker gene *Nr2f2* were observed between wild-type and *Etv2*^*enhΔ/Δ*^ embryos at E10.5 (Figure 2H). Co-immunostaining for the lymphatic endothelial marker Prox1 and the endothelial marker Erg1 at E10.5 showed overlapping expression, indicating that lymphatic endothelial cells were specified in *Etv2*^*enhΔ/Δ*^ embryos (Figures S2C and S2D). Taken together, these results demonstrate that early vascular development, including arterial, venous, and lymphatic specification, occur normally in *Etv2*^*enhΔ/Δ*^ hypomorphic mutants and suggest that 20% of *Etv2* expression is sufficient for early vascular development.

### *Etv2*^*enhΔ/Δ*^ compound heterozygotes display embryonic lethality at mid-gestation with evident anemia

Although early endothelial specification and vascular development appeared normal in *Etv2*^*enhΔ/Δ*^ mutants, as noted above, the reduction of *Etv2* expression to ~20% of the normal level resulted in embryonic lethality by E11.5. Indeed, *Etv2*^*enhΔ/Δ*^ compound heterozygous embryos appeared pale and anemic with a significant lack of evident blood cells in the yolk sac and embryo at E10 (Figure 2A). Similarly, *Etv2*^*enhΔ/Δ*^ mutant placentas were also pale and appeared anemic at E10.5, although they were morphologically normal (Figure S2E), and we considered the possibility that defects in the development of the placental vasculature might contribute to the observed lethality in *Etv2*^*enhΔ/Δ*^ mutant embryos. However, the vasculature in both wild-type and *Etv2*^*enhΔ/Δ*^ placentas appeared to develop normally, as evidenced by CD31 immunostaining of E10.5 placenta sections (Figure S2F), supporting the notion that the observed lethality in *Etv2*^*enhΔ/Δ*^ hypomorphic mutants was specific to blood development.

**Figure 3.**
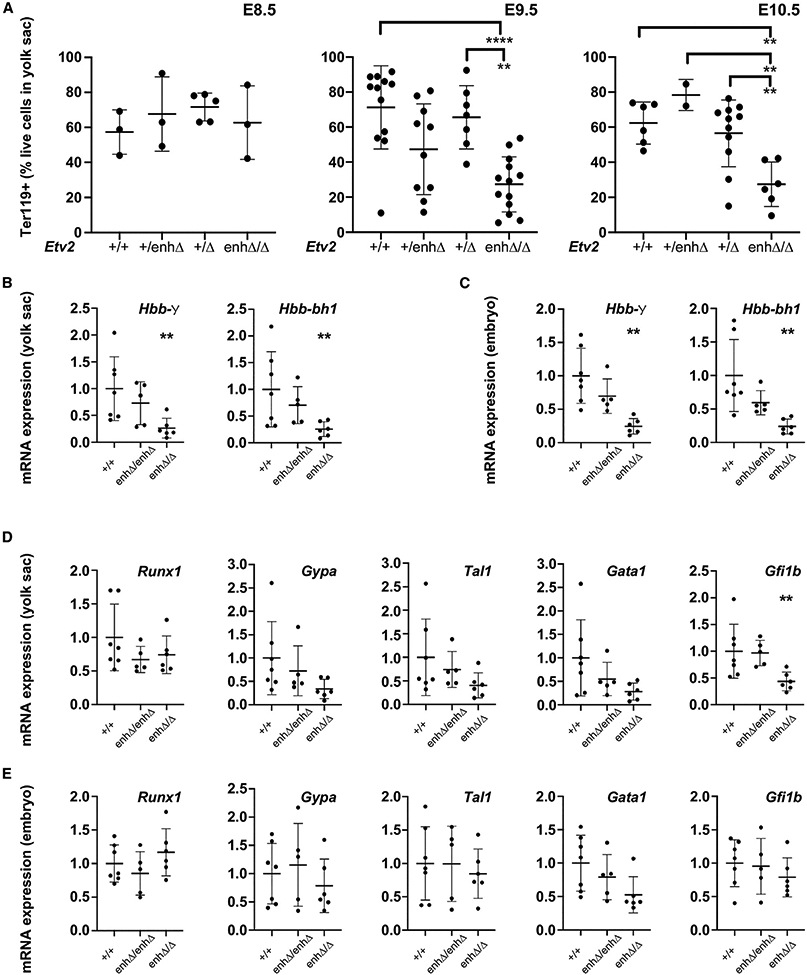
*Etv2*^*enhΔ/Δ*^ compound heterozygotes are anemic due to defects in erythropoietic development (A) FACS analysis expression of the erythropoietic marker Ter119 in cells isolated at E8.5 (left), E9.5 (middle), and E10.5 (right) from *Etv2*^*+/+*^, *Etv2*^*+/enhΔ*^, *Etv2*^*+/Δ*^, and *Etv2*^*enhΔ/Δ*^ yolk sacs. (B–E) qPCR analyses of erythropoietic markers *Hbb*-γ and *Hbb-bh1* in E9.5 yolk sacs (B) and embryos (C) and of hematopoietic markers *Runx1*, *Gypa, Tal1, Gata1*, and *Gfi1b* in *Etv2*^*+/+*^, *Etv2*^enhΔ/enhΔ^, and *Etv2*^*enhΔ/Δ*^ E9.5 yolk sacs (D) and E9.5 embryos (E). **p < 0.01, ****p <0.0001. Data are presented as mean ± SD and were analyzed by one-way ANOVA, followed by Bonferroni’s multiple comparison test. The number of biological samples analyzed for each genotype at each stage is indicated by individual data points on the graphs.

To confirm the visually apparent anemic phenotype in *Etv2*^*enhΔ/Δ*^ embryos, we measured the percentage of yolk sac cells expressing the erythrocyte marker Ter119 by flow cytometry at E8.5, E9.5, and E10.5 (Figures 3A and S3A). There was no significant difference in the number of Ter119+ cells in the yolk sac *Etv2*^*enhΔ/Δ*^ embryos compared with embryos of other genotypes at E8.5 (Figure 3A, left). However, at E9.5 and E10.5, *Etv2*^*enhΔ/Δ*^ yolk sacs had a significant decrease in the percentage of Ter119+ cells compared with control yolk sacs (Figure 3A, middle and right). To define the anemic phenotype in *Etv2*^*enhΔ/Δ*^ embryos in additional detail, we next examined molecular markers of early erythropoietic development in *Etv2*^*enhΔ/Δ*^ and control embryos at E9.5 by qPCR. Importantly, there was a significant decrease in the expression of several markers of erythropoiesis, including the embryonic hemoglobin markers *Hbb-bh1* and *Hbb-γ* in the embryo and yolk sac of *Etv2*^*enhΔ/Δ*^ hypomorphic mutants compared with wild-type and *Etv2*^*enhΔ/enhΔ*^ embryos and yolk sac (Figures 3B and 3C). Likewise, at E9.5, *Etv2*^*enhΔ/Δ*^ yolk sacs had significantly reduced expression of the erythropoietic transcription factor gene *Gfi1b* and also showed a trend (p < 0.1) toward reduced expression of the early erythropoietic genes *Gata1* and *Gypa* (Figures 3D and 3E).

To examine whether the hemogenic endothelium in the yolk sac was correctly specified at E9.5, we quantified hemogenic endothelial cells by performing flow cytometry for CD31+ and CD117+ cells (Fang et al., 2016; Hirschi, 2012). We observed an increase in the percentage of CD31-CD117 double-positive hemogenic endothelial cells in the yolk sacs of *Etv2*^*enhΔ/Δ*^ mutants compared with wild-type yolk sacs (Figure S2B). Likewise, whole-mount immunostaining of E10.5 yolk sacs for Lyve1, a recently identified marker of hemogenic endothelium specifically within the yolk sac (Lee et al., 2016), showed that the hemogenic endothelium was specified and properly patterned in the yolk sac of *Etv2*^*enhΔ/Δ*^ mutants (Figure S2A).

We further examined the E10.5 yolk sac for the presence of hematopoietic progenitor cells by performing FACS analysis with the hematopoietic marker CD45. Interestingly, we observed a slight increase in CD45+ cells in *Etv2*^*enhΔ/Δ*^ mutants (Figure S3B). To determine if downstream hematopoietic lineages were also affected, we looked for the presence of CD45+/CD11b+ myeloid progenitor cells, and CD45+/CD11b−/CD93+ lymphoid progenitor cells (McGrath et al., 2015). While both myeloid and lymphoid cells comprise only a small proportion of the hematopoietic niche at this stage, FACS analysis for these markers revealed a slight increase in the percentage of myeloid cells but not lymphoid cells in *Etv2*^*enhΔ/Δ*^ mutant yolk sacs (Figure S3B). These results suggest that hemogenic endothelial cells, as well as hematopoietic progenitors, are specified in *Etv2*^*enhΔ/Δ*^ mutants, but in the absence of normal erythropoietic development, hematopoietic progenitors either fail to progress from their progenitor state or perhaps contribute aberrantly to myeloid or other lineages.

To determine whether definitive hematopoiesis in the embryo was perturbed in *Etv2*^*enhΔ/Δ*^ mutants, we examined the AGM region for the presence of hemogenic endothelium at E10.5 (Figure S3C). As noted above, arterial specification, marked by Sox17 expression, occurred normally in this region of *Etv2*^*enhΔ/Δ*^ mutants (Figures 2G and S3C). However, Runx1 expression was substantially reduced in the AGM region of *Etv2*^*enhΔ/Δ*^ mutants compared with wild-type embryos (Figure S3C). Consistent with this observation, Runx1+ hematopoietic stem cell clusters were readily apparent in wild-type embryos but were less prevalent in *Etv2*^*enhΔ/Δ*^ mutants (Figure S3C, white arrows).

Taken together, these results demonstrate impaired primitive and definitive erythropoietic development in *Etv2*^*enhΔ/Δ*^ mutants. Moreover, these results establish that whereas ~20% of *Etv2* expression is sufficient to support vascular endothelial development, this level of *Etv2* expression is inadequate for proper blood development, and that the resultant anemic phenotype is likely the cause of the mid-gestational lethality in *Etv2*^*enhΔ/Δ*^ embryos.

### Down-regulation of the erythropoietic gene expression program in *Etv2*^*enhΔ/Δ*^ mutants

To further define the erythropoietic defects in *Etv2*^*enhΔ/Δ*^ embryos and to gain insight into the differential requirement for Etv2 in blood and vascular development, we sought to define changes in gene expression in *Etv2* mutants on a genome-wide scale. Therefore, we performed RNA-sequencing (RNA-seq) of yolk sac RNA from E8.5 embryos across all *Etv2* genotypes (*Etv2*^*+/+*^, *Etv2*^*+/enhΔ*^, *Etv2*^*+/Δ*^, *Etv2*^*enhΔ/enhΔ*^, *Etv2*^*enhΔ/Δ*^, and *Etv2*^*Δ/Δ*^) in our allelic series, which represents a gradient of *Etv2* expression ranging from 100% to 0% of normal expression, as shown in Figures 1B and 1C. We examined RNA expression in the E8.5 yolk sac, since the erythropoietic defect was not evident in *Etv2*^*enhΔ/Δ*^ mutant embryos at that stage (Figure 3A), and we reasoned that assaying the transcriptome at E8.5 would identify early, primary molecular changes responsible for defective blood development in *Etv2*^*enhΔ/Δ*^ mutants.

**Figure 4.**
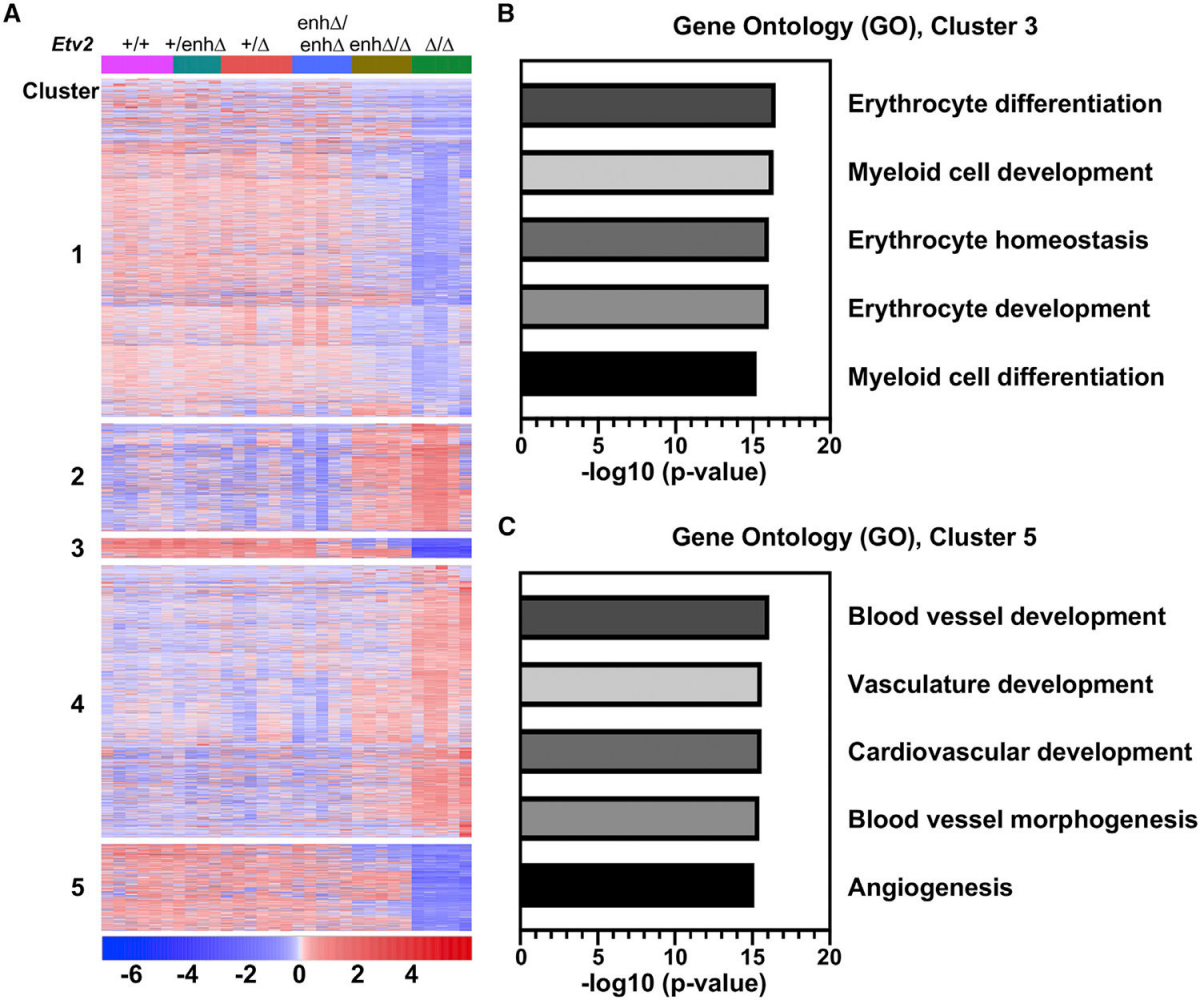
Down-regulation of the erythropoietic gene expression program in *Etv2*^*enhΔ/Δ*^ mutants (A) Heatmap representation of significantly differentially expressed genes from E8.5 yolk sacs across different genotypes, indicated at the top, of the *Etv2* allelic series. Differentially expressed genes clustered into five groups based on their expression patterns across each of the six genotypes such that clusters 1, 3, and 5 primarily consist of down-regulated genes in *Etv2*^*enhΔ/Δ*^ and *Etv2*^*Δ/Δ*^ yolk sacs, whereas clusters 2 and 4 mostly consist of genes up-regulated in *Etv2*^*enhΔ/Δ*^ and *Etv2*^*Δ/Δ*^ yolk sacs. The number and sex of biological samples used for RNA-seq analyses is indicated by colored shapes in the PCA plot in Figure S4A. (B and C) Top-scoring gene ontology (GO) analyses ranked in order of p value for differentially expressed genes in cluster 3 (B) and cluster 5 (C).

Differential gene expression analysis revealed that ~3,000 genes were changed (FDR < 0.05). Roughly one-half of the genes with significantly changed expression were down-regulated in either the null (*Etv2*^*Δ/Δ*^) mutants, the compound heterozygous (*Etv2*^*enhΔ/Δ*^) mutants, or both groups (Table S1). Principal-component analysis (PCA) clearly showed that whereas the heterozygous mutant yolk sacs clustered closely with wild-type yolk sacs, each of the mutant groups (*Etv2*^*enhΔ/Δ*^ and *Etv2*^*Δ/Δ*^) clustered separately (Figure S4A). Additionally, more genes were significantly down-regulated than were up-regulated in both mutant groups (Figures S4B and S4C), consistent with a role for Etv2 primarily as a transcriptional activator. Analysis of the gene expression data by HOPACH (hierarchical ordered partitioning and collapsing hybrid) clustering (van der Laan and Pollard, 2003) revealed that the differentially expressed genes clustered into five broad groups (Figure 4A).

Among the five clusters of differentially regulated genes (Figure 4A), genes in clusters 1, 2, and 4 were minimally changed between *Etv2*^*enhΔ/Δ*^ and *Etv2*^*Δ/Δ*^, and the gene ontology (GO) terms associated with those clusters did not suggest a likely role in either vascular or erythropoietic development (Figures 4A and S4D-S4F). Interestingly, GO terms associated with cluster 4, representing genes primarily up-regulated in *Etv2*^*enhΔ/Δ*^ and *Etv2*^*Δ/Δ*^ mutants, suggest roles in heart development (Figure S4F), consistent with earlier studies suggesting a role for Etv2 in suppressing cardiac development (Liu et al., 2012; Rasmussen et al., 2011). In contrast, cluster 3 and cluster 5 encompassed many genes with profoundly reduced expression in either *Etv2*^*Δ/Δ*^-null or *Etv2*^*enhΔ/Δ*^ hypomorphic mutants or both, and GO terms associated with those clusters suggested important roles for those groups of genes in vascular and hematopoietic development (Figure 4).

Cluster 3 included genes with significantly and profoundly reduced expression in both *Etv2*^*Δ/Δ*^-null and *Etv2*^*enhΔ/Δ*^ hypomorphic mutants, while cluster 5 included genes that were reduced in expression primarily in *Etv2*^*Δ/Δ*^-null mutants (Figure 4A). GO analyses revealed that the differentially expressed genes in cluster 3 are primarily associated with blood development, whereas the differentially expressed genes in cluster 5 are associated with vascular development. Indeed, the most significantly associated GO terms for genes in cluster 3 and cluster 5 were non-overlapping. The most significant GO terms for cluster 3 genes were erythrocyte differentiation, myeloid cell development, erythrocyte homeostasis, erythrocyte development, and myeloid cell differentiation (Figure 4B). The most significant GO terms for cluster 5 genes were blood vessel development, vasculature development, cardiovascular development, blood vessel morphogenesis, and angiogenesis (Figure 4C). Taken together, these RNA-seq data demonstrate distinct gene expression phenotypes in *Etv2*^*enhΔ/Δ*^ hypomorphic and *Etv2*^*Δ/Δ*^-null mutants that are consistent with the subsequent anemia phenotype in the hypomorphs and the complete loss of vascular and blood development in the nulls. In addition, these data provide support for the notion that erythropoietic genes are more sensitive to the reduced *Etv2* expression in hypomorphic *Etv2*^*enhΔ/Δ*^ mutants than are endothelial genes, which appear to be expressed at near wild-type level with only 20% of normal *Etv2* expression.

### Erythroid, but not endothelial, genes are highly sensitive to *Etv2* expression level

**Figure 5.**
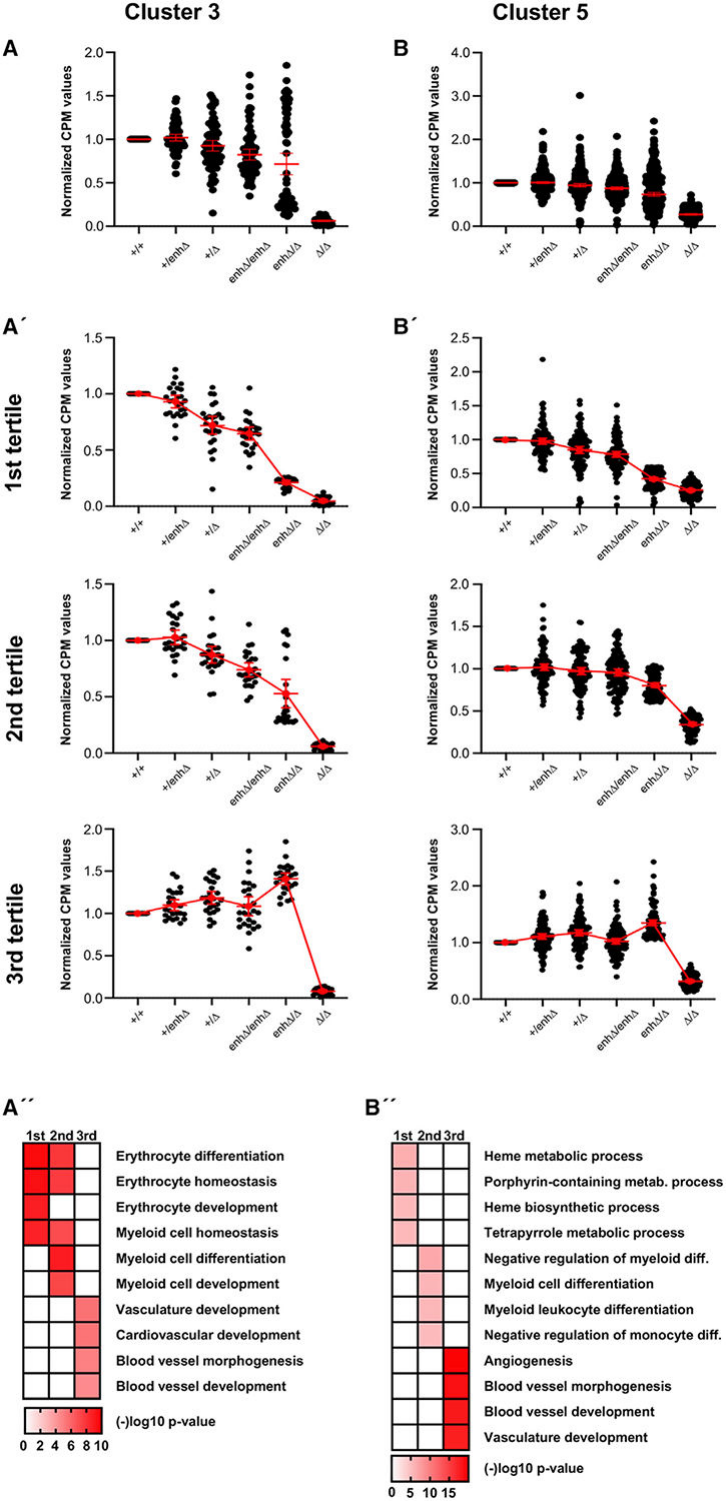
Erythropoietic genes are highly sensitive to a threshold of *Etv2* expression (A and B) Normalized expression for each differentially expressed gene in cluster 3 (A) and cluster 5 (B) plotted across all six *Etv2* genotypes (+/+, +/enhΔ, +/Δ, enhΔ/enhΔ, enhΔ/Δ, and Δ/Δ), where each dot represents a unique gene. (A′ and B′) Stratification of differentially expressed genes by tertile based on descending, ranked expression in each cluster across all six *Etv2* genotypes. (A″ and B″) Top-scoring GO terms for differentially expressed genes in each tertile ranked in order of p value and represented as a heatmap for cluster 3 (A″) and cluster 5 (B″). Lower (more significant) p values are shown in darker red; scale for −log10 p value is shown at the bottom of each heatmap. Tertiles are indicated at the top of each heatmap. Data are presented as mean ± 95% CI.

To gain additional insight into the erythropoietic and endothelial transcriptional programs regulated by Etv2, we plotted the normalized expression for each differentially expressed gene in cluster 3 (erythropoietic cluster) and cluster 5 (endothelial cluster) for each different *Etv2* genotype in our allelic series (Figures 5A and 5B and Table S2). These analyses showed that the complete loss of *Etv2* expression in *Etv2*^*Δ/Δ*^ mutants resulted in significant reduction in the expression of most genes in cluster 3 and cluster 5 (Figures 5A and 5B). In contrast, reduction of Etv2 expression by 80% in *Etv2*^*enhΔ/Δ*^ hypomorphic mutants appeared to result in two distinct sub-clusters for cluster 3 genes, with one sub-cluster exhibiting wild-type or greater levels of expression and a second sub-cluster showing dramatically reduced expression, similar to the expression of those genes observed in *Etv2*^*Δ/Δ*^-null mutants (Figure 5A). Cluster 5 genes exhibited a wide, uniform spread of expression levels in *Etv2*^*enhΔ/Δ*^ hypomorphic mutants, ranging from high to low expression and resembling the pattern of gene expression level observed in allelic combinations that result in ~50% of wild-type *Etv2* expression (Figure 5B).

To further characterize genes most affected by reduced *Etv2* expression, we next ranked the genes in each cluster on the basis of their expression level in *Etv2*^*enhΔ/Δ*^ mutants, divided each cluster into tertiles, and plotted each gene’s ranked expression across the different *Etv2* genotypes (Figures 5A′ and 5B′). These analyses revealed that genes in the first tertile, particularly from the first tertile of cluster 3, are highly sensitive to the dosage of Etv2 such that they show substantially reduced expression (>80% loss of expression) in *Etv2*^*enhΔ/Δ*^ mutants, similar to the expression levels observed for those same genes in Etv2-null mutants (Figures 5A′ and 5B′, first tertile). In contrast, genes in the third tertile were essentially insensitive to Etv2 dosage such that those genes were expressed at wild-type levels across all *Etv2* genotypes, except *Etv2*^*Δ/Δ*^ mutants, where complete loss of *Etv2* resulted in profound reduction in expression (Figures 5A′ and 5B′, third tertile). GO analyses of the tertiles revealed that the Etv2 dose-sensitive genes in the first tertiles of clusters 3 and 5 are largely involved in erythropoietic development, whereas the Etv2 dose-insensitive genes in the third tertiles are primarily associated with endothelial and vascular development (Figures 5A″ and 5B″). Together, these analyses indicate that the erythropoietic and endothelial gene programs are differentially sensitive to *Etv2* expression level. The endothelial program is resistant to loss of *Etv2* expression—20% of wild-type expression is sufficient to support normal expression of most genes in the endothelial program. In contrast, 20% of normal *Etv2* expression is not sufficient to support expression of genes in the erythropoietic program.

### Etv2 directly regulates the endothelial GRN and indirectly regulates the erythropoietic GRN

**Figure 6.**
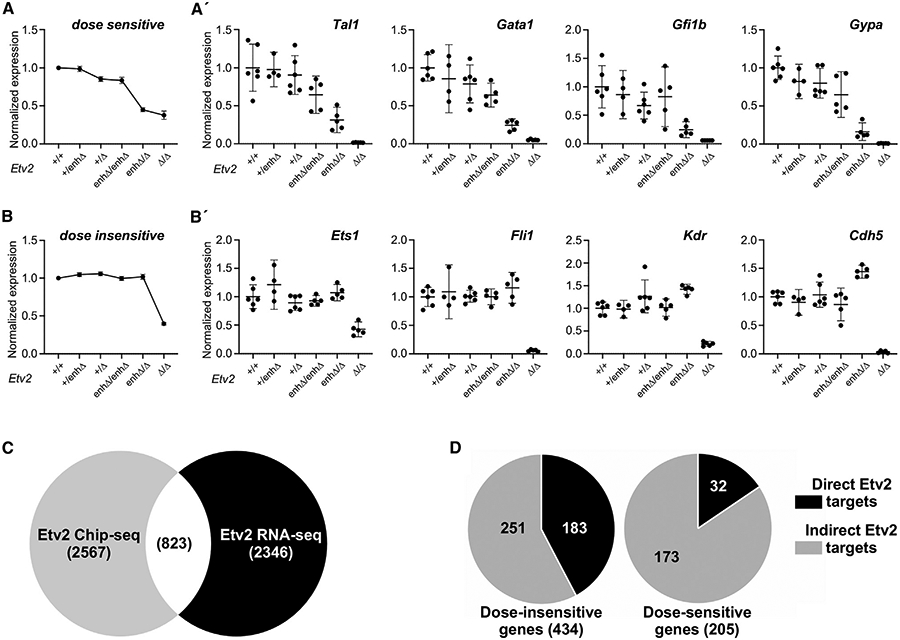
Dose-sensitive erythropoietic genes are indirect targets of Etv2 (A and B) Plots of normalized mean expression of *Etv2* dose-sensitive (A) and dose-insensitive (B) differentially expressed genes in E8.5 yolk sacs across all six *Etv2* genotypes. Plots in (A′) show normalized expression across all *Etv2* genotypes for selected dose-sensitive genes, including key erythropoietic genes *Tal1, Gata1, Gfi1b*, and *Gypa*. Plots in (B′) show normalized expression across all *Etv2* genotypes for selected dose-insensitive endothelial genes *Ets1, Fli1, Kdr*, and *Cdh5*. The number of biological samples for each genotype is indicated by individual data points in each graph. Data are presented as mean ± 95% CI. (C) Venn diagram of the intersection of Etv2 ChIP-seq (Liu et al., 2015) and RNA-seq datasets shows that 823 of 3,169 differentially expressed genes are direct targets of Etv2. (D) Intersection of Etv2 dose-sensitive and dose-insensitive genes with analysis of direct Etv2 targets reveals that 42.2% of all Etv2 dose-insensitive genes (183/ 434) are direct targets of Etv2, whereas only 15.6% of Etv2 dose-sensitive genes (32/205) are direct Etv2 targets.

Our RNA-seq data strongly support the idea that Etv2-dependent genes fall into two broad categories: Etv2 dose sensitive and Etv2 dose insensitive. Etv2 dose-sensitive genes exhibit significantly reduced expression in *Etv2*^*enhΔ/Δ*^ hypomorphic mutants to a degree similar to the reduction observed *Etv2*^*Δ/Δ*^ mutants. This pattern was evident when we plotted the mean expression values of all genes in this category across all *Etv2* genotypes (Figure 6A and Table S2). In contrast, Etv2 dose-insensitive genes exhibited reduced expression only in *Etv2*^*Δ/Δ*^ complete-null mutants, whereas they showed wild-type expression level in *Etv2*^*enhΔ/Δ*^ hypomorphs (Figure 6B and Table S2). Examples of dose-sensitive genes included key factors involved in erythropoietic development, such as *Tal1, Gata1, Gfi1b*, and the erythropoietic membrane protein *Gypa* ( Figure 6A′). Conversely, critical genes involved in endothelial development, including Ets transcription factors *Fli1* and *Ets1* and canonical markers of endothelial fate such as *Kdr* and *Cdh5*, were encompassed in the Etv2 dose-insensitive category ( Figure 6B′ ).

Given Etv2’s role as a non-redundant master regulator of embryonic endothelial and hematopoietic development (Koyano-Nakagawa and Garry, 2017; Lammerts van Bueren and Black, 2012; Sumanas and Choi, 2016), we were surprised to identify such an obvious differential requirement for Etv2 in these two related developmental programs. We reasoned that the molecular basis underlying the dose sensitivity of erythropoietic genes compared with endothelial genes might be reflected in direct versus indirect regulation by Etv2. As a first test of this idea, we intersected all differentially expressed genes from our RNA-seq analysis with a previously published Etv2 ChIP-seq dataset from an analysis performed on *in vitro* differentiated hemato-endothelial progenitors (Liu et al., 2015; Figure 6C and Table S3). Based on the overlap with ChIP-seq peaks, 823 of 3,169 differentially expressed genes across the six *Etv2* genotypes appear to be direct targets of Etv2 (Figure 6C). Interestingly, 42.2% of dose-insensitive genes are direct targets of Etv2, compared with only 15.6% of dose-sensitive genes (Figure 6D). Indeed, the odds ratio of a dose-insensitive gene being a direct Etv2 target is 4.3 times greater than the odds of a dose-sensitive gene being a direct target. These results strongly support the notion that a substantial fraction of genes involved in endothelial development are direct targets of Etv2, whereas only a small percentage of erythropoietic genes appear to be direct targets of Etv2. Instead, erythropoietic genes that are dependent on Etv2 are predominantly indirect targets and also appear to be far more sensitive to perturbations in the level of Etv2 expression.

### Etv2 regulates erythropoietic genes indirectly via Tal1 and Gata1

The observation that the genes encoding the essential hematopoietic transcription factors Tal1 and Gata1 were dose-sensitive targets of Etv2 (Figure 6A′) suggested that Etv2 might indirectly regulate the erythropoietic program through Tal1 and Gata1 as downstream effectors. As an initial test of this idea, we intersected our Etv2 dose-sensitive gene set with previously published Tal1 and Gata1 ChIP-seq datasets (Goode et al., 2016; Kassouf et al., 2010; Yu et al., 2009). We found that 33% (69/205) of all dose-sensitive Etv2 target genes were directly bound by both Tal1 and Gata1 (Figure S5A), suggesting co-regulation by these two factors downstream of Etv2. In contrast, only 18% (79/434) of dose-insensitive genes were directly co-regulated by Gata1 and Tal1 (Figure S5A′). Moreover, network interaction analysis with Tal1 and Gata1 as central factors identified that 42 of the 69 co-regulated, dose-sensitive genes are part of the network involved in erythropoietic development (Figure S5B).

Next, we examined the loci of several markers of erythropoietic development, including the *Tal1* and *Gata1* genes themselves, for Tal1- and Gata1-binding sites previously identified in ChIP-seq experiments (Goode et al., 2016; Figures S6A and S6B). Interestingly, both *Tal1* and *Gata1* are auto- and co-regulated by each other; i.e., Tal1 regulates its own gene and the *Gata1* gene, and likewise, Gata1 binds its own gene and the *Tal1* gene (Figures S6A and S6B). Similarly, the genes encoding the transcription factor *Gfi1b* and the erythropoietin receptor gene *Epor*, two genes essential for erythropoiesis (Kieran et al., 1996; Lin et al., 1996; Vassen et al., 2014), are also bound directly by Tal1 and Gata1 (Figures S6C and S6D). Interestingly, only *Tal1*, and not *Gata1*, is a direct target of Etv2 (Liu et al., 2015; Figure 6C).

**Figure 7.**
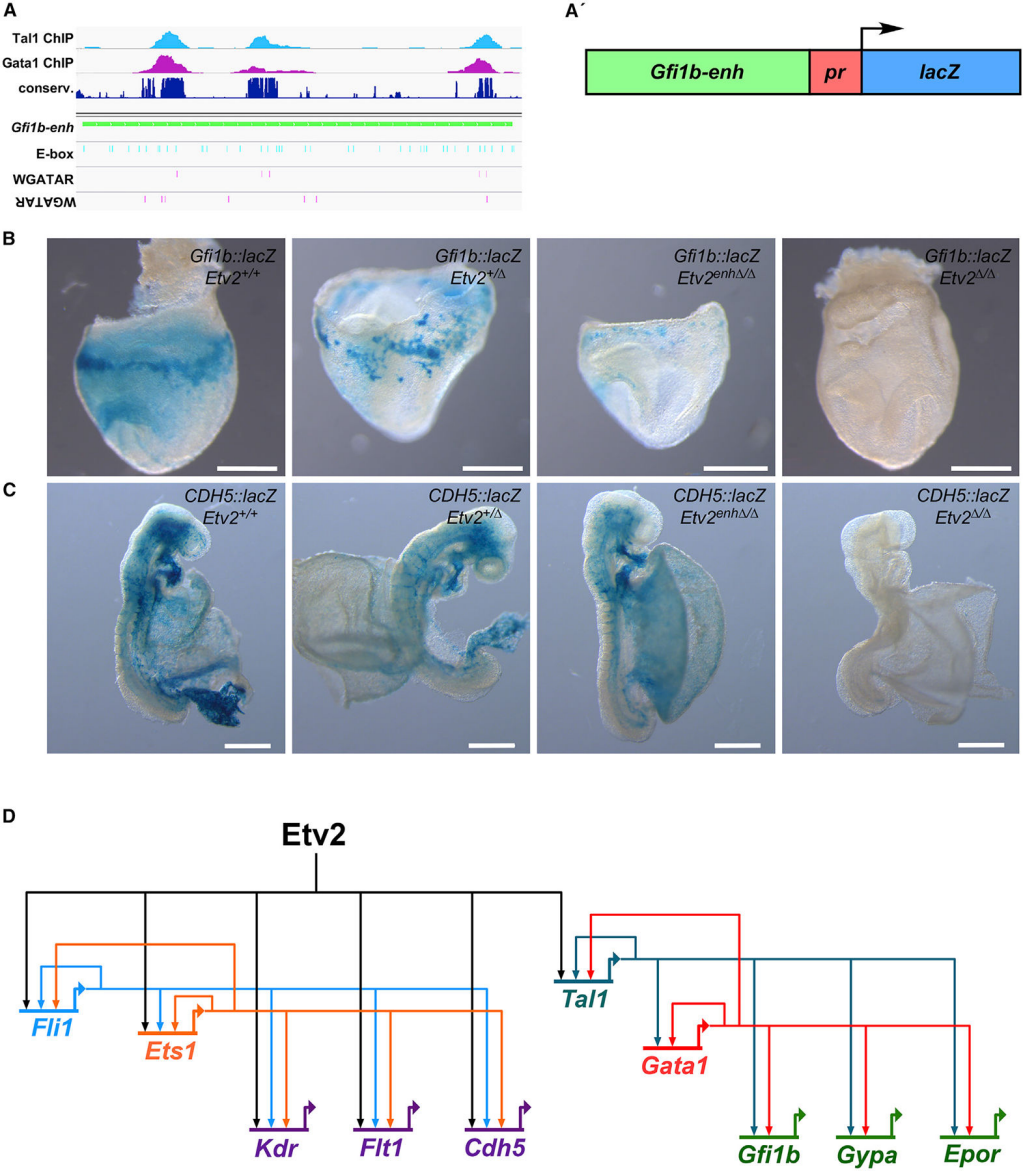
A 3′-enhancer of *Gfi1b* is an indirectly regulated and highly dose-sensitive Etv2 target (A) Browser track views showing Tal1 (light blue peaks) and Gata1 (purple peaks) occupancy at a 3′ enhancer of *Gfi1b* (green bar). Dark blue peaks show conservation across 60 vertebrate species. Note increased conservation at regions also bound by Tal1 and Gata1. Blue and purple vertical lines indicate the presence of E-boxes and GATA-binding motifs (WGATAR) in the *Gfi1b* enhancer locus. (A′) Schematic depiction of the *Gfi1b* transgene used to create *Gfi1b*::*lacZ* transgenic lines. (B) X-gal staining of E8.0 *Gfi1b*::*lacZ* transgenic embryos on *Etv2*^*+/+*^, *Etv2*^*+/Δ*^, *Etv2*^*enhΔ/Δ*^, and *Etv2*^*Δ/Δ*^ backgrounds. Note the nearly absent reporter activity in the *Gfi1b*::*lacZ*;*Etv2*^*enhΔ/Δ*^ embryo. (C) X-gal staining of E8.5 *CDH5*::*lacZ* transgenic embryos on *Etv2*^*+/+*^, *Etv2*^*+/Δ*^, *Etv2*^*enhΔ/Δ*^, and *Etv2*^*Δ/Δ*^ backgrounds. Note the robust reporter activity in the *CDH5*::*lacZ*;*Etv2*^*enhΔ/Δ*^ embryo, similar to activity observed on *Etv2*^*+/+*^, *Etv2*^*+/Δ*^, and *Etv2*^*enhΔ/Δ*^ backgrounds. Scale bars, 500 μm. For (B) and (C), a minimum of three embryos/genotype were analyzed; representative images are shown. (D) Model gene-regulatory network (GRN) for an Etv2-initiated coherent feedforward loop for early yolk sac endothelial and erythropoietic development. In this model, Etv2 directly activates endothelial genes to initiate endothelial development (left). In contrast, Etv2 indirectly activates the erythropoietic GRN (right) via activation of a single transcription factor, *Tal1*. Tal1 then serves as the direct regulator of the erythropoietic feedforward circuit via autoregulation and positive-feedback activation of *Gata1*.

Taken together with our RNA-seq and phenotypic data, these ChIP-seq data suggest that Tal1 and Gata1 function in a hierarchical fashion downstream of Etv2, wherein Etv2 directly activates Tal1, which in turn activates itself and Gata1 to drive erythropoietic development (Figure 7D).

Given the central role of Tal1 in early erythropoiesis as a downstream effector of Etv2, we also examined the spatial expression pattern of *Tal1* during development at E8.5 and E10.5 in *Etv2*^*enhΔ/Δ*^ compound heterozygotes (Figure S7). Importantly, we found that *Tal1* expression was essentially reduced in a uniform manner in the yolk sac and embryo at E8.5 and E10.5 in *Etv2*^*enhΔ/Δ*^ hypomorphic mutants, suggesting that the defective erythropoiesis in *Etv2*^*enhΔ/Δ*^ mutants occurs due to a general sensitivity of *Tal1* expression to Etv2 dose rather than due to loss in a specific embryonic compartment.

### A *Gfi1b* enhancer is an indirect and highly dosage-sensitive Etv2 target

As an explicit test of the idea that Etv2 regulates erythropoietic gene expression indirectly in a dose-dependent manner, we sought to examine an enhancer of an indirect, dose-sensitive target of Etv2 to determine how it would respond to the level of Etv2 expression in *Etv2* hypomorphic and-null mutants compared with a known direct Etv2 target enhancer. A previously described enhancer of *Gfi1b*, sufficient to direct expression to hematopoietic lineages, contains multiple Tal1- and Gata1-binding sites but lacks detectable Etv2 binding by ChIP (Figures 7A and S6C; Moignard et al., 2013). We cloned this enhancer fragment into a *hsp68-lacZ* transgenic reporter plasmid (Figure 7A′) and generated *Gfi1b*::*lacZ* transgenic mice. X-gal staining of transgenic embryos revealed reporter activity largely in the blood-forming islands of the embryonic yolk sac at E8.0 (Figure 7B, *Etv2*^*+/+*^). When the *Gfi1b*::*lacZ* enhancer transgene was crossed onto *Etv2*^*+/+*^, *Etv2*^*+/Δ*^, *Etv2*^*enhΔ/Δ*^, and *Etv2*^*Δ/Δ*^ backgrounds, we observed strong β-galactosidase activity in blood-forming regions at E8.0 only on *Etv2*^*+/+*^ and *Etv2*^*+/Δ*^ backgrounds (Figure 7B). Importantly, β-galactosidase activity from the *Gfi1b*::*lacZ* enhancer transgene was nearly absent in the presence of 20% of normal Etv2 expression on the *Etv2*^*enhΔ/Δ*^ background and was entirely lost in *Etv2*^*Δ/Δ*^-null embryos (Figure 7B). Conversely, when we crossed a *CDH5*::*lacZ* enhancer transgene, which is known to be a direct Etv2 target (De Val et al., 2008), onto the same *Etv2* mutant backgrounds, we observed loss of β-galactosidase activity at E8.5 only in the absence of *Etv2* expression in *Etv2*-null embryos (Figure 7C). In the presence of 20% of *Etv2* expression in *Etv2*^*enhΔ/Δ*^ hypomorphic embryos, the *CDH5*::*lacZ* enhancer transgene exhibited strong activity, similar to the level observed in *Etv2*^*+/+*^ and *Etv2*^*+/Δ*^ embryos (Figure 7C). Together, the results shown in Figures 6 and 7 demonstrate that erythropoietic enhancers are highly sensitive to Etv2 dose compared with endothelial enhancers.

## DISCUSSION

GRNs that specify cell and tissue fates are initiated by master regulatory factors that activate downstream transcriptional cascades, most often involving feedforward and positive-feedback loops, which serve to reinforce the network and determine cellular identity (Peter and Davidson, 2017). Activating, coherent feedforward circuits are commonly used kernels in developmental GRNs. They are defined as transcriptional circuits in which an upstream regulator, often a master regulatory factor, activates downstream target genes in the network, including other transcription factors, which in turn activate the same downstream genes in the network (Peter and Davidson, 2017). Positive-feedback circuits involve transcription factors activating their own expression and serve to reinforce and stabilize GRNs (Peter and Davidson, 2017).

Etv2 is a prime example of a master regulatory factor, functioning in a feedforward fashion at the top of the hemato-endothelial GRN (Garry, 2016; Lammerts van Bueren and Black, 2012; Sumanas and Choi, 2016). Previous work has shown that an initial pulse of *Etv2* expression at ~E7.5 in hemato-endothelial progenitors in the early mouse mesoderm initiates both the endothelial and the hematopoietic programs, and then *Etv2* expression rapidly turns off and by E9.5 is largely extinguished (Ferdous et al., 2009; Lee et al., 2008). Under normal circumstances this early pulse of Etv2 is sufficient to induce the coherent feedforward GRNs for both endothelial and erythropoietic development, which are reliant on downstream transcription factors such as Fli1 and Ets1 in the case of endothelial development and Tal1 and Gata1 in the case of erythropoietic development (Figure 7D). However, our data presented here suggest that the details for how Etv2 induces these two GRNs are different.

In the case of the endothelial program, Etv2 functions directly in the feedforward endothelial GRN, regulating several early intermediate transcription factors, including *Fli1* and *Ets1*, and also directly regulating many downstream differentiation genes required for endothelial function, such as VEGF receptors *Kdr* and *Flt1*, and canonical cell surface marker genes, such as *Cdh5* (Figures 6B′ and 7D). Because the downstream effectors of Etv2 in the endothelial GRN, Fli1 and Ets1, are not dose sensitive ( Figure 6B′), and because Etv2 functions directly in the endothelial GRN feedforward circuit, reduction of *Etv2* to 20% of normal expression does not disrupt the endothelial program, and vascular development proceeds normally in *Etv2*^*enhΔ/Δ*^ hypomorphic mutants. In contrast, the erythropoietic feedforward GRN is activated by Etv2 only via Tal1, and it is Tal1, rather than Etv2, that serves as the primary feedforward regulator of the erythropoietic GRN (Figure 7D). Indeed, among the early erythropoietic transcription factor genes downstream of Etv2, only Tal1 appears to be a direct target and, importantly, is also a dose-sensitive Etv2 target ( Figure 6A′). Thus, our model suggests that Tal1 functions downstream of Etv2 as a second master regulator for erythropoietic development. Consistent with this notion, Tal1 is sufficient to rescue hematopoietic development *in vitro* in Etv2-null murine embryonic stem cells (Wareing et al., 2012). However, the Etv2 dose sensitivity of *Tal1* renders it susceptible to a threshold effect such that reduction of *Etv2* expression below a certain level fails to sufficiently activate the Tal1-dependent feedforward and positive-feedback circuits that are essential for erythropoietic development (Figure 7D), and erythropoietic development fails in *Etv2*^*enhΔ/Δ*^ hypomorphic mutants. In support of this idea, an elegant study of hematopoietic differentiation *in vitro* demonstrated that a substantially higher threshold of *Etv2* is required to initiate *Tal1* expression and in turn transactivate the downstream hemogenic differentiation program (Zhao and Choi, 2017).

The feedforward function of Tal1 in the erythropoietic GRN depends, at least in part, on the activation of the transcription factor Gata1, and together, Tal1 and Gata1 function as key co-regulators of the downstream GRN (Wadman et al., 1997; Wu et al., 2014; Figure 7D). Consistent with this notion, a significant fraction of dose-dependent, indirect Etv2 targets in the erythropoietic GRN have direct binding sites for Tal1 and Gata1 in their enhancers (Figure S5A). Moreover, network interaction analysis shows a direct connection between numerous key erythropoietic genes with either Tal1 or Gata1 or both, supporting the roles for these two transcription factors as the central nodes in the erythropoietic network downstream of Etv2 (Figures S5B and 7D).

Homozygous deletion of the 1.4-kb upstream enhancer described here resulted in an ~60% reduction in *Etv2* expression *in vivo* (Figures 1B and 1C), indicating clearly that this element functions as a *bona fide Etv2* enhancer. On the other hand, the fact that homozygous deletion of the enhancer did not completely abolish *Etv2* expression indicates that other redundant or shadow enhancers governing early *Etv2* expression must also exist (Osterwalder et al., 2018). The observation that *Etv2*^*enhΔ/enhΔ*^ homozygous enhancer deletion mice were viable and apparently normal is noteworthy because it indicates that deletion of the enhancer does not result in the complete loss of *Etv2* expression from any required, specific temporal or spatial pattern. Rather, these observations support the notion that deletion of the enhancer primarily affected the level of *Etv2* expression, creating a hypomorphic allele, as discussed here. In addition, the observation that deletion of the *Etv2* enhancer from the mouse genome resulted in embryonic lethality only when combined in *trans* with an *Etv2*-null allele suggests that the enhancer is not required for the expression of genes other than *Etv2*. If the enhancer were required for the expression of other neighboring or distal genes, then homozygous deletion of the enhancer would be expected to have a more profound effect on those genes than the *trans*-heterozygous allelic combination present in *Etv2*^*enhΔ/Δ*^ mice, where only one copy of the enhancer is deleted.

Interestingly, the same proximal upstream enhancer described here was previously deleted from the mouse genome as part of a larger deletion that also removed the *Etv2* proximal promoter from the genome (Koyano-Nakagawa et al., 2015). Unsurprisingly, that larger, 3.9-kb *Etv2* deletion resulted in a complete loss of *Etv2* expression and early embryonic demise, mimicking the Etv2-null phenotype (Koyano-Nakagawa et al., 2015), presumably due to deletion of the *Etv2* promoter and transcriptional start site. In contrast, in the work here, only the enhancer was deleted, leaving the promoter and transcriptional start site intact and revealing the likely existence of additional *Etv2* enhancers. This type of enhancer redundancy is common in genes encoding master regulators and is proposed to provide robustness during development (Kvon et al., 2021; Osterwalder et al., 2018). Ultimately, it will be interesting to determine how multiple enhancers function coordinately to control the level and pattern of *Etv2* expression *in vivo*.

### Limitations of the study

In this study, we show that the early endothelial and erythropoietic gene regulatory networks in the developing embryo are highly sensitive to the dosage of the master hemato-endothelial *tran*-scription factor, Etv2. Although the hemogenic endothelium and hematopoietic precursors in the yolk sac of *Etv2*^*enhΔ/Δ*^ mutants appear to be properly specified, whether these progenitors retain their ability to differentiate into all hematopoietic lineages remains to be determined. Interestingly, the hemogenic endothelium in the AGM region of *Etv2*^*enhΔ/Δ*^ mutant embryos shows a decrease in the expression of Runx1 and a profound reduction in the presence of hematopoietic clusters at E10.5. However, since most *Etv2*^*enhΔ/Δ*^ embryos are significantly anemic and close to demise by this stage, it remains to be determined whether the embryonic hematopoietic phenotype in these mutants is a direct result of the decrease in Etv2 expression or secondary to the anemia phenotype, which is evident by this stage. Finally, how Etv2 dosage directly affects *Tal1* expression through its action on specific *Tal1* enhancers remains to be determined.

## STAR★METHODS

### RESOURCE AVAILABILITY

#### Lead contact

Further information and requests for resources and reagents should be directed to Brian Black, PhD (brian.black@ucsf.edu).

#### Materials availability

Mouse lines generated in this study will be made available upon request.

#### Data and code availability

The RNA-seq dataset generated in this study has been deposited in the GEO with accession number GSE174546 and will be publicly available as of the date of publication. This paper analyzes existing, publicly available data. The accession numbers for these datasets are listed in the key resources table. Raw data used to generate the microscopy images will be made available by the lead contact upon request.

This paper does not report original code.

Any additional information required to reanalyze the data reported in this paper is available from the lead contact upon request.

### EXPERIMENTAL MODEL AND SUBJECT DETAILS

#### Transgenic and knockout mouse models

*Etv2*::*lacZ*, *Etv2enh*::*lacZ*, and *Gf1b*::*lacZ* transgenic mice were generated by pronuclear microinjection using standard methods, as previously described (De Val et al., 2004). To generate transgene constructs, fragments from mouse genomic DNA (genomic coordinates in Key resources table) were amplified by PCR and cloned into p-AUG-β-gal (for *Etv2*::*lacZ*) or *hsp68-lacZ* (for *Etv2enh*::*lacZ*, and *Gf1b*::*lacZ*) transgenic vectors (De Val et al., 2008). Transgenes were released by digestion with appropriate restriction enzymes, purified using the Qiagen gel extraction kit (cat# 28,704) and diluted to 2ng/μL prior to injection. Transgenic founders were either collected for transient transgenic analyses or used to establish stable transgenic lines. *CDH5*::*lacZ* transgenic mice direct β-galactosidase expression under the control of a 3564-bp promoter and enhancer fragment of the human *CDH5* gene and were previously described as *VE-CADHERIN*::*lacZ* transgenic mice (De Val et al., 2008). *Etv2*^*Δ/+*^ mice were generated by crossing *Etv2*^*flox/flox*^ males (Lee et al., 2011) to *Mef2c-AHF-Cre* females, resulting in Cre-dependent deletion of floxed exons 4-5, which encode the DNA binding domain, in all cells of F1 offspring, due to Cre expression in the female germline (Ehlers et al., 2014; schematic in Figure 1A). Intercrosses between *Etv2*^*Δ/+*^ mice resulted in *Etv2*^*Δ/Δ*^ embryos, which phenocopied previously characterized *Etv2*^*−/−*^ embryos (Ferdous et al., 2009; Thompson et al., 1993).

*Etv2*^*+/enhΔ*^ mice were generated by CRISPR-mediated genome editing as described previously (Anderson et al., 2017). Briefly, pairs of single guide RNAs (sgRNAs) targeting genomic sequence 5′ and −3′ of the 1.4-kb *Etv2* enhancer described here were designed using CHOPCHOP45 (Labun et al., 2019) with upstream sgRNA: 5′-agtatttgattacgaagtcc-3′ and downstream sgRNA: 5′-gtacacaccgcaagtccaca-3′. Knockout mice were engineered using a mix containing Cas9 mRNA (100 ng/μL) and two sgRNAs (25 ng/μL each) in injection buffer (10 mM Tris, 0.1 mM EDTA, pH 7.5). This mix was injected into the cytoplasm of fertilized FVB mouse zygotes. Generation 0 (F0) founder mice were genotyped using PCR with High Fidelity Platinum Taq Polymerase (Thermo Fisher Scientific) to identify those founders with the desired non-homologous end-joining (NHEJ)-generated deletion breakpoints. Sanger sequencing was used to identify and confirm deletion breakpoints in F0 and F1 mice. Two founder lines for the *Etv2*^*enhΔ*^ allele were established by independently crossing with wild type mice, and the *Etv2*^*enhΔ/Δ*^ compound heterozygous phenotype was observed to be substantially identical in both lines.

All mouse lines were maintained on a mixed background and all animal experiments performed at UCSF were reviewed and approved by the UCSF Institutional Animal Care and Use Committee. All animal work performed at Lawrence Berkeley National Laboratory (LBNL) was reviewed and approved by the LBNL Animal Welfare and Research Committee. Female and male mice between 6 and 40 weeks of age and of appropriate genotypes were intercrossed to obtain control and mutant embryos of both sexes. Embryos were collected at developmental stages ranging from E7.0 to E13.5, as indicated in the figures and figure legends. Genotyping was performed on DNA isolated from yolk sacs or from tail biopsies by PCR (primer sequences in Table S4).

### METHOD DETAILS

#### Embryo collection, X-gal staining, *in situ* hybridization, and immunostaining

The day of the plug was designated as E0.5 and embryos of different stages from E7.0 to E13.5 were collected and processed appropriately for different experiments. X-gal staining, Salmon-gal (S-gal) staining, *in situ* hybridization, and section immunostaining were carried out according to standard protocols (Anderson et al., 2004; Materna et al., 2019; Sinha et al., 2015; Wilkinson and Nieto, 1993). Primary and secondary antibodies are listed in the Key resources table and were used at a dilution of 1:100 and 1:500, respectively. The *in situ* hybridization probe for *Etv2* was amplified from mouse cDNA using primers 5′-ttatggatccCTTTCCAGGCGGAGCCCG-3′ and 5′-atgtgaattcCACCTCTTTGGGGTCGC-3′ and was then cloned in BamHI/EcoRI in pBluescript II SK+. T7 and T3 RNA polymerases were used to synthesize anti-sense probe and sense probes, respectively. RNAscope *in situ* hybridization was performed as previously described (de Soysa et al., 2019). The RNAScope probe for *Tal1* was purchased from Advanced Cell Diagnostics (ACD Bio 428221). Whole-mount immunofluorescence was performed as previously described (Sinha et al., 2015).

#### Image acquisition, analysis, and adjustment

Bright field and whole mount embryo images were captured on a Leica MZ165 FC stereoscope equipped with a DFC450 camera using the LAS software. Confocal imaging for visualizing vascular development and hematopoietic clusters in embryo sections was performed on an upright Leica SPE confocal scanning microscope and images were captured using the LAS software. Images were subsequently analyzed using ImageJ (Fiji) software. Confocal imaging of placental vasculature and of whole mount *Tal1 in situ* hybridized embryos was performed on an upright Zeiss LSM-780 confocal point scanning microscope and on an inverted Zeiss LSM-700 confocal point scanning microscope, respectively. Images were captured using the Zen Black software. All images were subsequently analyzed using ImageJ (Fiji) software. All images were compiled and linearly adjusted for brightness, contrast, and color balance using Adobe Photoshop.

#### Quantitative real-time, reverse transcriptase PCR (qPCR)

Somite-matched embryos and yolk sacs were dissected in diethyl pyrocarbonate (DEPC)-treated 1 × phosphate-buffered saline, and a small piece of the yolk sac was collected for genotyping. Embryos and yolk sacs were collected individually and lysed in Trizol-LS (Thermo Fisher Scientific). Total RNA was isolated according to manufacturer’s instructions and resuspended in nuclease free water. An equal amount of RNA for each sample was used for cDNA synthesis using the Quantitect Reverse Transcription kit (Qiagen). cDNA samples were then subjected to quantitative RT-PCR (qPCR) using the Maxima SYBR green system and a 7900HT Fast Real Time PCR System or a Quant Studio 5 Real Time PCR System (Applied Biosystems) with the primers listed in Table S4. Data were normalized to *Actb* (β-actin) expression by the 2^−ΔΔCt^ method (Schachterle et al., 2012).

#### Flow cytometric analyses and cell sorting

Whole embryos or yolk sacs were dissociated into a single cell suspension by passing through a 22-gauge needle in Hank’s Balanced Salt Solution with 5% fetal bovine serum and 1% penicillin/streptomycin and were subsequently incubated with a Collagenase II (Worthington)/DNAseI (Roche) solution (1 mg/mL) at 37°C for 30 min. Single cell suspensions were then stained with a 1:100 dilution of Rat anti-mouse Ter119-PerCP or with a mix of rat anti-mouse CD45-PercP, rat anti-mouse CD11b-Pacific Blue, and rat anti-mouse CD93 at a 1:20 dilution (see Key resources table) for 45 min at 4°C with agitation and were then subjected to either flow cytometric analyses on a BD FACS Verse or BD FACS Aria III flow cytometer (BD Biosciences). Flow cytometric data were analyzed using the FlowJo software (v10.0.7 – Tree Star; BD Biosciences).

#### RNA-sequencing and analyses

To prepare libraries for RNA-sequencing, matings were set up among *Etv2*^*+/enhΔ*^ and *Etv2*^*+/Δ*^ mice to obtain embryos for the following genotypes (*Etv2*^*+/+*^; *Etv2*^*+/enhΔ*^; *Etv2*^*+/Δ*^; *Etv2*^*enhΔ/enhΔ*^; *Etv2*^*enhΔ/Δ*^; *Etv2*^*Δ/Δ*^). Embryos were collected at E8.5, yolk sacs were immediately frozen in liquid nitrogen and stored at −80°C, and embryos were used for genotyping. Somite-matched embryos from each genotype were used for subsequent RNA extraction and cDNA library preparation and sequencing. RNA was extracted using the RNAeasy Plus Micro Kit from Qiagen (cat# 74034) and was analyzed for purity and quantity using the Agilent 2100 Bioanalyzer instrument. 10 ng of RNAfrom each sample was used to prepare cDNA libraries using the Trio RNA-seq kit from Tecan Biosciences (0507-96). cDNA libraries were pooled in 20 μL at a final concentration of 10 nM for each library, and then the pooled libraries were sequenced on the Illumina Hi-seq 4000 sequencer at the UCSF Center for Advanced Technologies. Data were returned as FastQ files. Trimming of known adapters and low-quality regions was performed using fastq-mcf (Aronesty, 2013). Sequence quality control was assessed using FastQC (https://www.bioinformatics.babraham.ac.uk/projects/fastqc/) and RSeQC (Wang et al., 2012). Alignment of the provided samples to the reference genome (mouse mm9) was performed using STAR 2.5.2a (Dobin et al., 2013). Reads were assigned to genes using the featureCounts (Liao et al., 2014), part of the Subread suite (http://subread.sourceforge.net/, and gene-level counts were arrived at using Ensembl gene annotation in GTF format.

p values for differential expression were calculated using edgeR, and genes with detectable expression were normalized using the calcNormFactors function in edgeR (Robinson et al., 2010; Robinson and Oshlack, 2010). Mean gene expression was modeled as a function of batch of processing (two batches), sex (male or female) and genotype (five different genotypes). Genes with expression associated with genotype were determined by the likelihood ratio test implemented in EdgeR. The built-in R function *p.adjust* was used to calculate the false discovery rate (FDR) for each p value using the Benjamini-Hochberg method (Benjamini and Hochberg, 1995). The expression of the genes that were associated with genotype with an FDR <0.05 was first clustered using the HOPACH package and then visualized using the pheatmap package (https://cran.r-project.org/package=pheatmap; van der Laan and Pollard, 2003). Genes from each of the HOPACH clusters were extracted and gene ontology (GO) analyses were performed using Metascape (Zhou et al., 2019).

Normalized counts per million (CPM) values for genes in clusters 3 and 5 were calculated from the log10 CPM values for each gene across all the samples. The CPM values were then normalized for all samples across each genotype and expressed as a ratio of gene expression in the wild type samples to calculate the relative CPM value for each gene.

#### ChIP-seq analyses and odds ratio calculation

Bed files for ChIP-seq datasets were downloaded from the GEO omnibus or from the associated paper.

For a previously published Etv2 ChIP-seq dataset (Liu et al., 2015), the consensus Etv2 bound peaks were determined using the union of replicate concordant peaks for each of the antibodies using the bedops program. The union was computed using the *bedops–merge* option while the replicate concordant peaks were computed using the *bedops –element-of* option. Each of the consensus peaks was linked to genes with the GREAT program (great.stanford.edu) using the default parameter settings associating genes with genomic locations. All (~18,000) genes used in the bulk RNA-seq experiment were associated with the input data to determine whether they were endothelial (dose-insensitive) or blood (dose-sensitive) genes (from the GREAT analyses). The change in the log odds (or odds ratio) of a dose-insensitive gene linked to at least one of the Etv2-bound enhancers versus the odds of a dose-sensitive gene linked to at least one of the Etv2-bound enhancers was estimated using the glm function in R with family = “binomial” setting.

Gata1 and Tal1-Chip-seq datasets from hematopoietic progenitors (Goode et al., 2016; GEO GSE69101), Gata1 ChIP-seq from mouse erythroleukemia cells (Yu et al., 2009; GEO GSE16594), and Tal1 Chip-seq from E12.5 fetal erythrocytes (Kassouf et al., 2010) were downloaded as bed files. The nearest genes associated with each peak were extracted using the GREAT tool (McLean et al., 2010). The gene lists were then intersected with the appropriate differentially expressed gene lists from our RNA-seq analyses and Venn diagrams were plotted using the Eulerr application (https://cran.r-project.org/package=eulerr). The 3-way Venn diagram was plotted using http://bioinformatics.psb.ugent.be/webtools/Venn/. Cytoscape (Shannon et al., 2003) was used to visualize the protein interaction network for the 69 dose-sensitive genes from the 3-way Venn diagram. The STRING database (Szklarczyk et al., 2019) was used as a source for these interactions where the edges represent association based on common function or co-expression with a medium confidence threshold of 0.4. Browser tracks for ChiP-seq datasets were visualized using the Integrative Genomics Viewer (Robinson et al., 2011).

### QUANTIFICATION AND STATISTICAL ANALYSIS

3-5 biological replicates per condition were used for all experiments subjected to quantitative analysis (FACS analyses, qRT-PCR analyses, RNA-seq analyses, and immunostaining). Statistical analyses were performed, and graphs were generated by using the GraphPad Prism (v7.03) software package. Data were analyzed by one-way ANOVA followed by Bonferroni’s multiple comparison test or by unpaired two-tailed, student’s t-test. Graphed results in Figures 1,2, 3, S2, and S3 are presented as mean ± standard deviation. Graphed results in Figures 4 and 5 are presented as mean value ± 095% confidence interval (CI). The n values for each condition and experiment presented in the graphs are depicted by an individual point. Details for statistical tests for each experiment can be found in the figure legends.

## Supplementary Material

1

2

3

4

5

## Figures and Tables

**Table:** KEY RESOURCES TABLE

REAGENT or RESOURCE	SOURCE	IDENTIFIER
Antibodies		
Rat anti-mouse CD31	BD Biosciences	RRID: AB_396660
Goat anti-human SOX17	R and D systems	RRID: AB_355060
Rabbit anti-mouse Runx1	Abcam	RRID: AB_2049267
Rabbit anti-mouse Erg	Abcam	RRID: AB_2630401
Goat anti-human PROX1	R and D systems	RRID: AB_2170716
Donkey anti-Rat 488	Thermo Fisher	RRID: AB_141709
Donkey anti-Goat 594	Thermo Fisher	RRID: AB_2534105
Rat anti-mouse TER119-PerCP	Biolegend	RRID: AB_893635
Rat anti-mouse CD93-PE	BD Biosciences	RRID: AB_397003
Rat anti-mouse CD45-PerCP	BD Biosciences	RRID: AB_396609
Rat anti-mouse CD11b-Pacific Blue	Thermo Fisher	RRID: AB_10372795
Rat anti-mouse CD117-FITC	BD Biosciences	RRID: AB_394805
Rat anti-mouse CD31-APC	BD Biosciences	RRID: AB_398497
Deposited data		
RNA seq data	This study	GEO: GSE174546
Etv2 ChIP seq data	Liu et al. (2015) PMID: 25802403	GEO: GSE59402
Gata1 ChIP seq data (MEL cell line)	Yu et al. (2009) PMID: 19941827	GEO: GSE16594
Tal1 ChIP seq (E12.5 Fetal liver erythroid cells)	Kassouf et al. (2010) PMID: 20566737	GEO: GSE18720
Gata1 and Tal1 ChIP seq data (Hematopoietic progenitors)	Goode et al. (2016) PMID: 26923725	GEO: GSE69101
Experimental models: Organisms/strains		
*Etv2Flox/Flox*	Lee et al. (2011) PMID: 21425416	MGI (ID): 5009246
*Mef2c-AHF-Cre*	Verzi et al. (2005) PMID: 16188249 Ehlers et al. (2014) PMID: 25242327	MGI (ID): 3639735
*Cdh5*::*lacZ*	De Val and Black (2009) PMID: 19070576	N/A
*Etv2 EnhΔ*	This manuscript	chr7:31,423,347-31,425,058 (mm9)
*Etv2*::*lacZ*	This manuscript	chr7:31,421,209-31,424,519 (mm9)
*Etv2enh*::*lacZ*	This manuscript	chr7:31,423,559-31,425,018 (mm9)
*Gfi1benh*:: *hsp68-lacZ*	This manuscript	chr2:28,452,623-28,458,241 (mm9)
Oligonucleotides		
Refer to Supplemental Table S2	Integrated DNA Technologies	N/A
Software and algorithms		
ImageJ	Schindelin et al. (2012) PMID: 22743772	https://imagej.nih.gov/ij/
Prism(v7.0)	Graphpad	https://www.graphpad.com/scientific-software/prism/
Venn diagram generator	N/A	http://bioinformatics.psb.ugent.be/webtools/Venn/
Integrative Genomics Viewer	Robinson et al. (2011) PMID: 21221095	https://software.broadinstitute.org/software/igv
ECR browser	Ovcharenko et al. (2004) PMID: 15215395	https://ecrbrowser.dcode.org/
EdgeR	Robinson et al. (2010) PMID: 19910308	https://bioconductor.org/packages/release/bioc/html/edgeR.html
HOPACH	van der Laan and Pollard (2003)	https://www.bioconductor.org/packages/release/bioc/html/hopach.html
pheatmap	Kolde (2019)	https://CRAN.R-project.org/package=pheatmap
GREAT	McLean et al. (2010) PMID: 20436461	http://great.stanford.edu/
Flow Jo (v10.0.7)	N/A	https://www.flowjo.com/
FastQC	Wang et al. (2012) PMID: 22743226	https://www.bioinformatics.babraham.ac.uk/projects/fastqc/)
STAR 2.5.2a	Dobin et al. (2013) PMID: 23104886	N/A
Subread suite	Liao et al. (2014) PMID: 24227677	http://subread.sourceforge.net/
Metascape	Zhou et al. (2019) PMID: 30944313	https://metascape.org/

## Highlights

- *Etv2* hypomorphic mutant mouse embryos have profound anemia and normal vasculature
- Higher *Etv2* expression is required for erythropoiesis than for vasculogenesis
- Etv2 directly regulates the embryonic endothelial gene-regulatory network (GRN)
- Etv2 indirectly regulates the embryonic erythropoietic GRN via Tal1
