# Supplementary material for: Differential Etv2 threshold requirement for endothelial and erythropoietic development
Source: Cell Rep. Author manuscript; Available in PMC 2022 Jun 16. (PMC9203129; doi:10.1016/j.celrep.2022.110881)
Supplement: 1 [file NIHMS1812256-supplement-1.pdf]

Cell Reports, Volume *39*

Supplemental information

Differential Etv2 threshold requirement for endothelial and erythropoietic development

Tanvi Sinha, Kelly Lammerts van Bueren, Diane E. Dickel, Ivana Zlatanova, Reuben Thomas, Carlos O. Lizama, Shan-Mei Xu, Ann C. Zovein, Kohta Ikegami, Ivan P. Moskowitz, Katherine S. Pollard, Len A. Pennacchio, and Brian L. Black

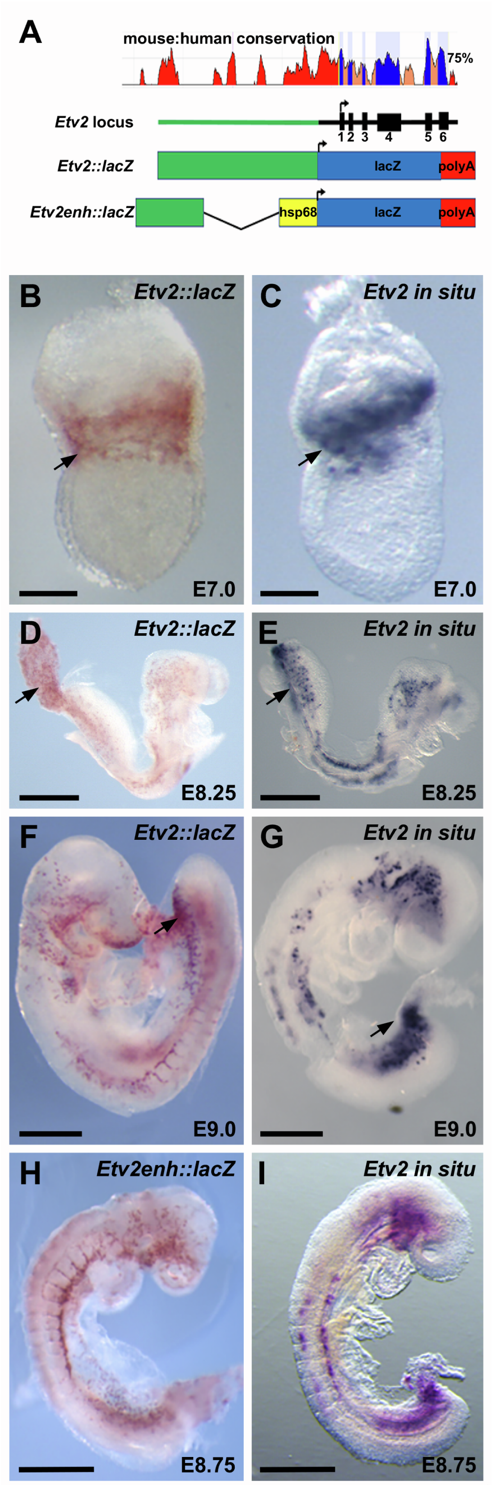
A
mouse:human conservation
75%
Etv2 locus
1 2 3 4 5 6
Etv2::lacZ
lacZ
polyA
Etv2enh::lacZ
hsp68
lacZ
polyA
B
Etv2::lacZ
E7.0
C
Etv2 in situ
E7.0
D
Etv2::lacZ
E8.25
E
Etv2 in situ
E8.25
F
Etv2::lacZ
E9.0
G
Etv2 in situ
E9.0
H
Etv2enh::lacZ
E8.75
I
Etv2 in situ
E8.75

Figure S1. An upstream *Etv2::lacZ* enhancer transgene recapitulates endogenous Etv2 expression. Related to Figure 1. (A) Top panel shows ECR browser tracks depicting mouse-human sequence conservation at the *Etv2* locus. The lower panels depict a schematic representation of the *Etv2* gene locus, a full length 3.3-kb Etv2 enhancer (*Etv2::lacZ*), and an upstream 1.4-kb *Etv2* enhancer (*Etv2enh::lacZ*) transgene. (B,D,F) Salmon-gal (S-gal) staining of F0 transgenic embryos showing that the full length 3.3-kb Etv2 enhancer (*Etv2::lacZ*) directs reporter expression in the developing hemato-vascular system (6 S-gal positive /10 transgene positive). S-gal staining was observed in the blood forming regions at E7.0 (arrow in B), in the angioblasts and the allantois (arrow in D) at E8.25 (D), and in the developing endothelial cells and inter-somitic vessels at E9.0 (F). This pattern recapitulates endogenous *Etv2* expression as observed by *in situ* hybridization for *Etv2* at E7.0 (C), E8.25 (E), and E9.0 (G). Arrowheads show selected regions of corresponding expression of *lacZ* reporter and endogenous *Etv2*. (H) S-gal staining of an *Etv2enh::lacZ* F0 transgenic embryo showing that a smaller upstream 1.4-kb *Etv2* enhancer fragment also directs reporter expression in the developing vascular system in a pattern that essentially recapitulates endogenous *Etv2* expression (I) (3 S-gal positive/5 transgene positive). (n=3 embryos/stage for *in situ* hybridization analysis). Scale bars: B,C, 200 μm; D-I, 500 μm.

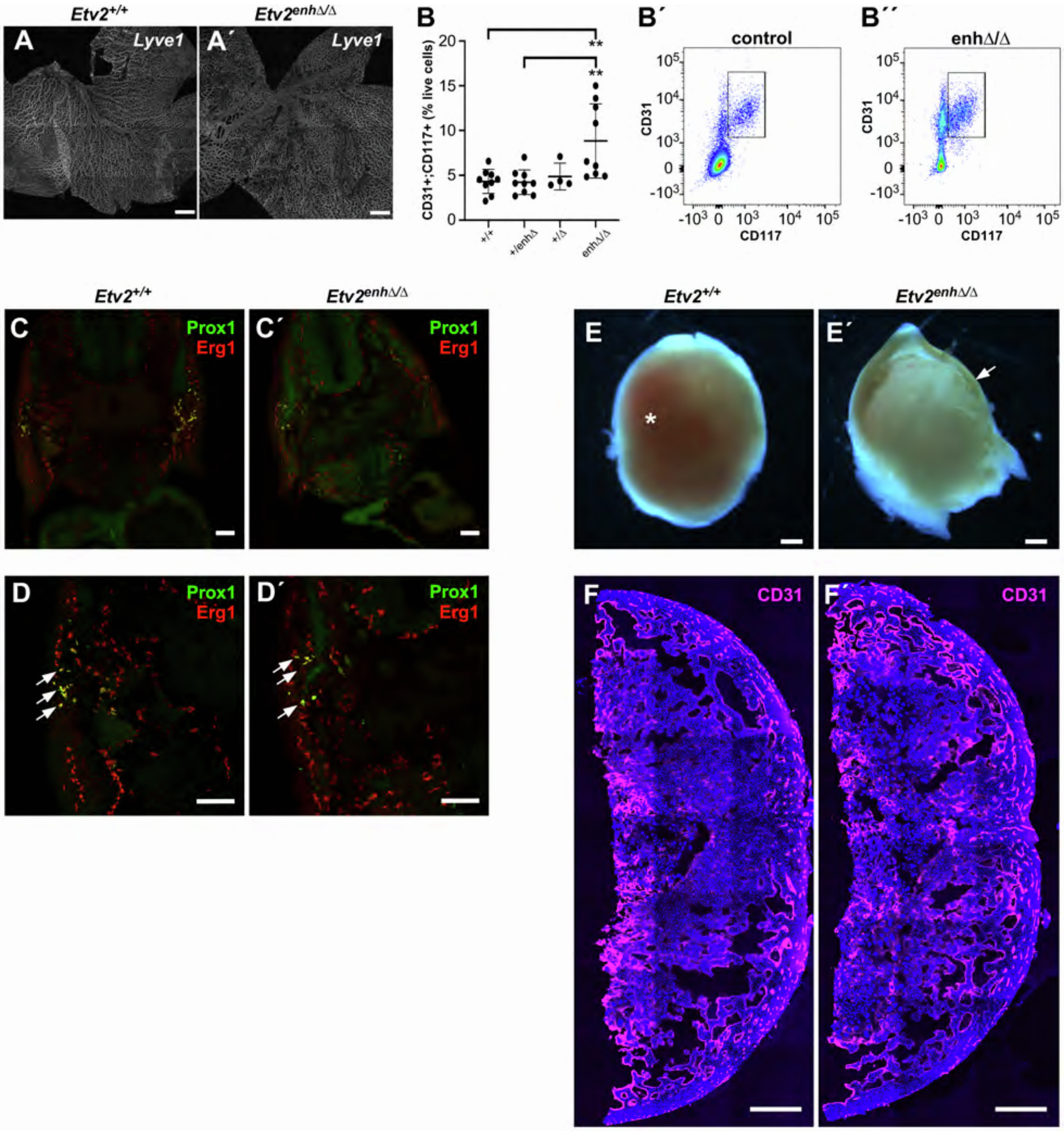

Etv2+/+
Etv2enhΔ/Δ
A
Lyve1
A´
Lyve1
B
CD31+;CD117+ (% live cells)
20
15
10
5
0
**
**
+/+
+/enhΔ
+/Δ
enhΔ/Δ
B´
control
B´´
enhΔ/Δ
CD31
CD117
C
C´
Prox1
Erg1
D
D´
E
E´
F
F´
CD31

Figure S2. *Etv2$^{enh\Delta/\Delta}$* compound heterozygotes exhibit apparently normal lymphatic and placental endothelial development. Related to Figure 2. (A) Images of whole mount E10.5 yolk sacs stained with the yolk sac hemogenic endothelial marker, Lyve1 (gray), showing similar levels and pattern of Lyve1 expression in *Etv2$^{+/+}$* (A) and *Etv2$^{enh\Delta/\Delta}$* (A′) yolk sacs. (B) Quantification and representative FACS plots of CD31+/CD117+ hemogenic endothelial cells in E9.5 control (B′) and *Etv2$^{enh\Delta/\Delta}$* (B′′) yolk sacs. The number of biological samples for each genotype is indicated by individual datapoints in each graph. Data are presented as mean ± SD and were analyzed by 1-way ANOVA, followed by Bonferroni's multiple comparison test. (C,D) Co-immunostaining of E10.5 embryo sections with Prox1 (green) and Erg1 (red) show the presence of lymphatic endothelial cells in both *Etv2$^{+/+}$* (C,D) and *Etv2$^{enh\Delta/\Delta}$* (C′,D′) embryos. Higher magnification images of C and C′ are shown in D and D′, respectively. White arrows indicate the presence of lymphatic endothelial cells. (E) Whole mount images of E10.5 placentas of *Etv2$^{+/+}$* (E) and *Etv2$^{enh\Delta/\Delta}$* (E′) embryos. White arrow in (E′) indicates the apparent lack of blood in the *Etv2$^{enh\Delta/\Delta}$* placenta. (F) Stitched images of placenta sections of *Etv2$^{+/+}$* (F) and *Etv2$^{enh\Delta/\Delta}$* (F′) embryos stained with endothelial marker, CD31 (magenta), and nuclear marker, DAPI (blue), show normal vascular development in *Etv2$^{enh\Delta/\Delta}$* placentas, similar to wild type embryos. Scale bars: A-A′, 500µm; C-D, 100µm; E-F, 500µm. For A,C-F, n = 3 biological replicates/genotype.

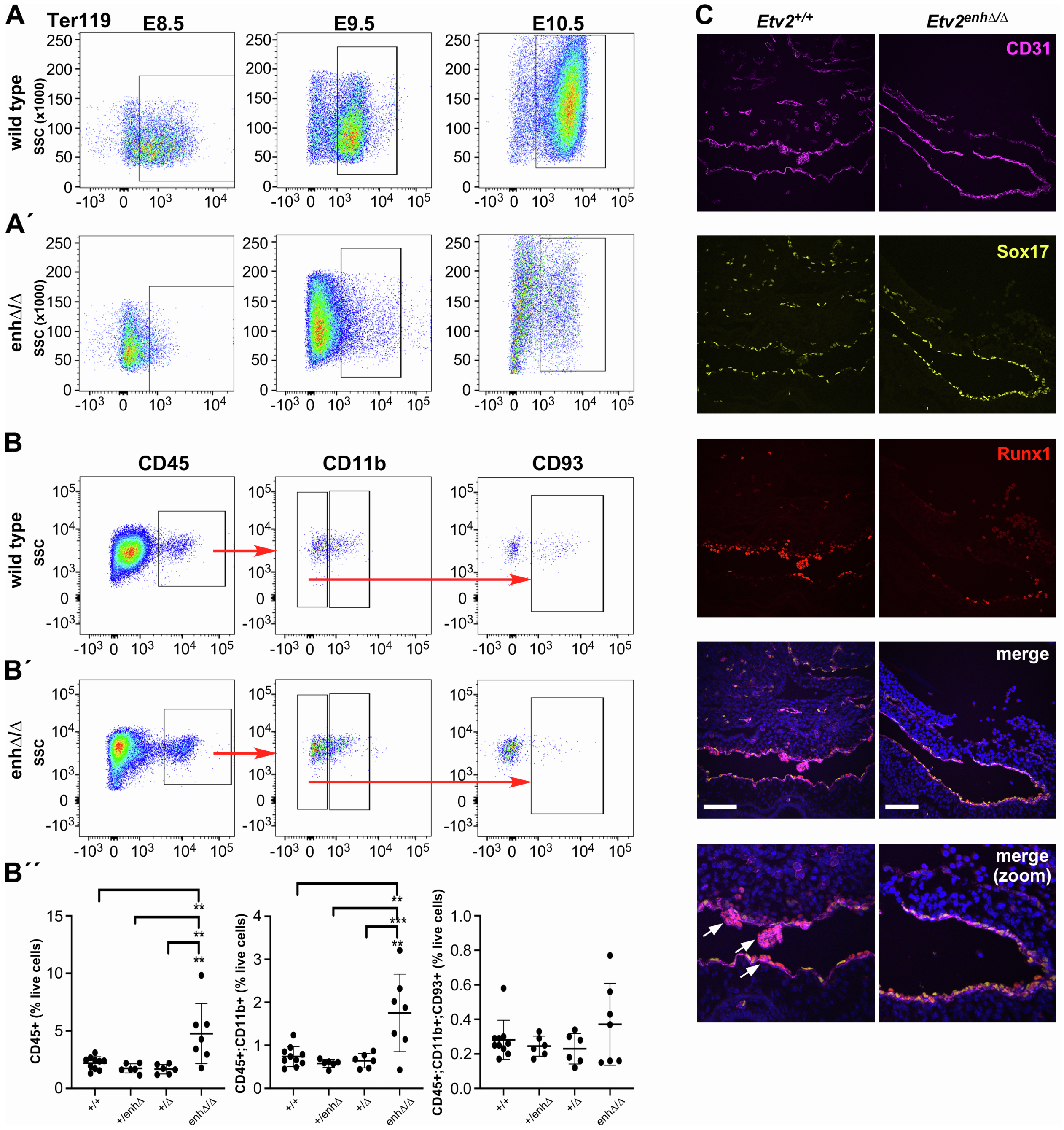

A
Ter119
E8.5
E9.5
E10.5
wild type
SSC (x1000)
A´
enhΔ/Δ
SSC (x1000)
B
CD45
CD11b
CD93
wild type
SSC
B´
enhΔ/Δ
SSC
B´´
CD45+ (% live cells)
CD45+;CD11b+ (% live cells)
CD45+;CD11b+;CD93+ (% live cells)
+/+
+/enhΔ
+/Δ
enhΔ/Δ
**
***
C
Etv2+/+
Etv2enhΔ/Δ
CD31
Sox17
Runx1
merge
merge (zoom)

Figure S3. ***Etv2***$^{enh\Delta/\Delta}$ **compound heterozygotes show defects in hematopoietic development. Related to Figure 3.** (A) FACS plots showing Ter119+ erythropoietic cells in wild type (A) and *Etv2*$^{enh\Delta/\Delta}$ (A′) yolk sacs at E8.5, E9.5, and E10.5. (B) FACS plots showing CD45+ hematopoietic cells, CD45+/CD11b+ myeloid cells, and CD45+/CD11b-/CD93+ lymphoid cells in wild type (B) and *Etv2*$^{enh\Delta/\Delta}$ (B′) yolk sacs at E10.5. Arrows in (B) and (B′) indicate gating strategies employed for quantification of myeloid and lymphoid cells. (B′′) Quantification of FACS data showing percentage of hematopoietic, myeloid, and lymphoid cells present in *Etv2*$^{+/+}$ (+/+), *Etv2*$^{+/enh\Delta}$ (+/enhΔ), *Etv2*$^{+/\Delta}$ (+/Δ), and *Etv2*$^{enh\Delta/\Delta}$ (enhΔ/Δ) yolk sacs. The number of biological samples for each genotype is indicated by individual datapoints in each graph. Data are presented as mean ± SD and were analyzed by 1-way ANOVA, followed by Bonferroni's multiple comparison test. (C) Co-immunostaining of the aorta-gonad-mesonephros region in E10.5 embryo sections with CD31 (magenta), Sox17 (yellow), and Runx1 (red), showing the presence of hematopoietic clusters in wild type (*Etv2*$^{+/+}$) but not in *Etv2*$^{enh\Delta/\Delta}$ embryos (arrows) (n=3 biological replicates/genotype). Blue, DAPI. Scale bars, 100µm.

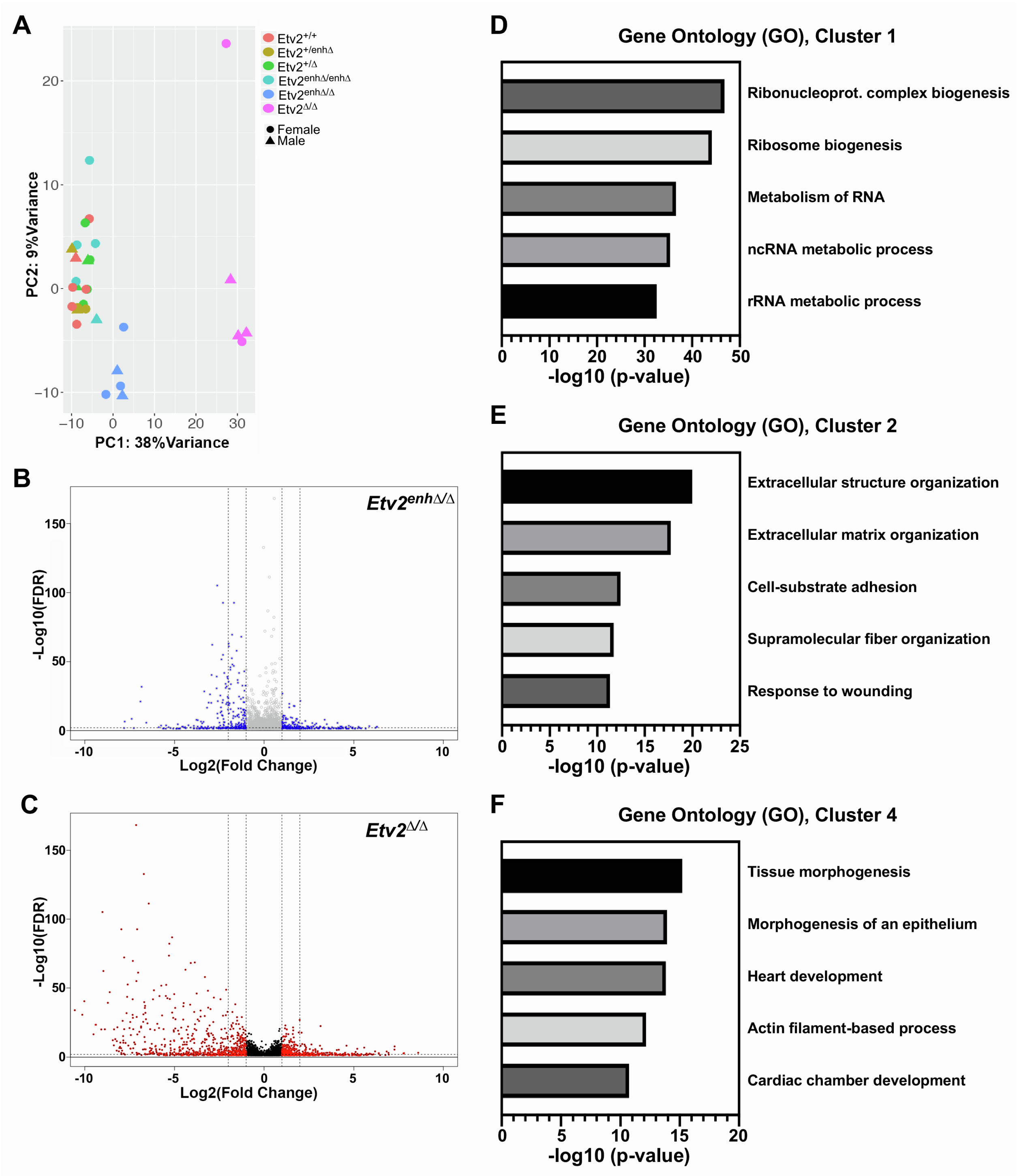

A
Etv2+/+
Etv2+/enhΔ
Etv2+/Δ
Etv2enhΔ/enhΔ
Etv2enhΔ/Δ
Etv2Δ/Δ
Female
Male
PC2: 9%Variance
PC1: 38%Variance
20
10
0
−10
−10
0
10
20
30
B
Etv2enhΔ/Δ
-Log10(FDR)
Log2(Fold Change)
150
100
50
0
-10
-5
0
5
10
C
Etv2Δ/Δ
-Log10(FDR)
Log2(Fold Change)
150
100
50
0
-10
-5
0
5
10
D
Gene Ontology (GO), Cluster 1
Ribonucleoprot. complex biogenesis
Ribosome biogenesis
Metabolism of RNA
ncRNA metabolic process
rRNA metabolic process
0
10
20
30
40
50
-log10 (p-value)
E
Gene Ontology (GO), Cluster 2
Extracellular structure organization
Extracellular matrix organization
Cell-substrate adhesion
Supramolecular fiber organization
Response to wounding
0
5
10
15
20
25
-log10 (p-value)
F
Gene Ontology (GO), Cluster 4
Tissue morphogenesis
Morphogenesis of an epithelium
Heart development
Actin filament-based process
Cardiac chamber development
0
5
10
15
20
-log10 (p-value)

Figure S4. Changes in gene expression in *Etv2*$^{enh\Delta/\Delta}$ and *Etv2*$^{\Delta/\Delta}$ yolk sacs revealed by RNA sequencing and gene ontology analysis of differentially expressed genes in clusters 1, 2, and 4,. Related to Figure 4. (A) Principal component analysis shows that embryonic yolk sacs from *Etv2*$^{+/e/nh\Delta}$, *Etv2*$^{+/\Delta}$, and *Etv2*$^{enh\Delta/enh\Delta}$ have similar gene expression profiles as wild type yolk sacs, evident by co-clustering. In contrast, *Etv2*$^{enh\Delta/\Delta}$ and *Etv2*$^{\Delta/\Delta}$ yolk sacs are significantly different from the wild type and heterozygous yolk sacs as well from each other along both principal component axes. No sex-based differences were observed in gene expression across all *Etv2* genotypes. The number and sex of biological samples used for RNA-seq analyses is indicated by colored shapes. (B,C) Volcano plots for differentially expressed genes in *Etv2*$^{enh\Delta/\Delta}$ (B) and *Etv2*$^{\Delta/\Delta}$ (C) yolk sacs depicting expression changes by at least 2-fold (-1<logFC>1) and FDR<0.05 in black and red dots respectively. These volcano plots also indicate that *Etv2*$^{\Delta/\Delta}$ yolk sacs show more profound changes in gene expression when compared to gene expression changes observed in *Etv2*$^{enh\Delta/\Delta}$ yolk sacs. Each dot on the volcano plot represents an individual gene. (D) GO analysis for differentially expressed genes in Cluster 1 of the HOPACH cluster showed that these genes were largely involved in general translational processes. Note these genes did not show profound changes in expression across the various *Etv2* genotypes. (E) Differentially expressed genes in Cluster 2, which were slightly upregulated in both *Etv2*$^{enh\Delta/\Delta}$ and *Etv2*$^{\Delta/\Delta}$ yolk sacs, are mainly associated with extracellular matrix organization and adhesion. (F) Differentially expressed genes in Cluster 4 were mainly up-regulated only in *Etv2*$^{\Delta/\Delta}$ yolk sacs and were largely involved in tissue morphogenesis and heart development.

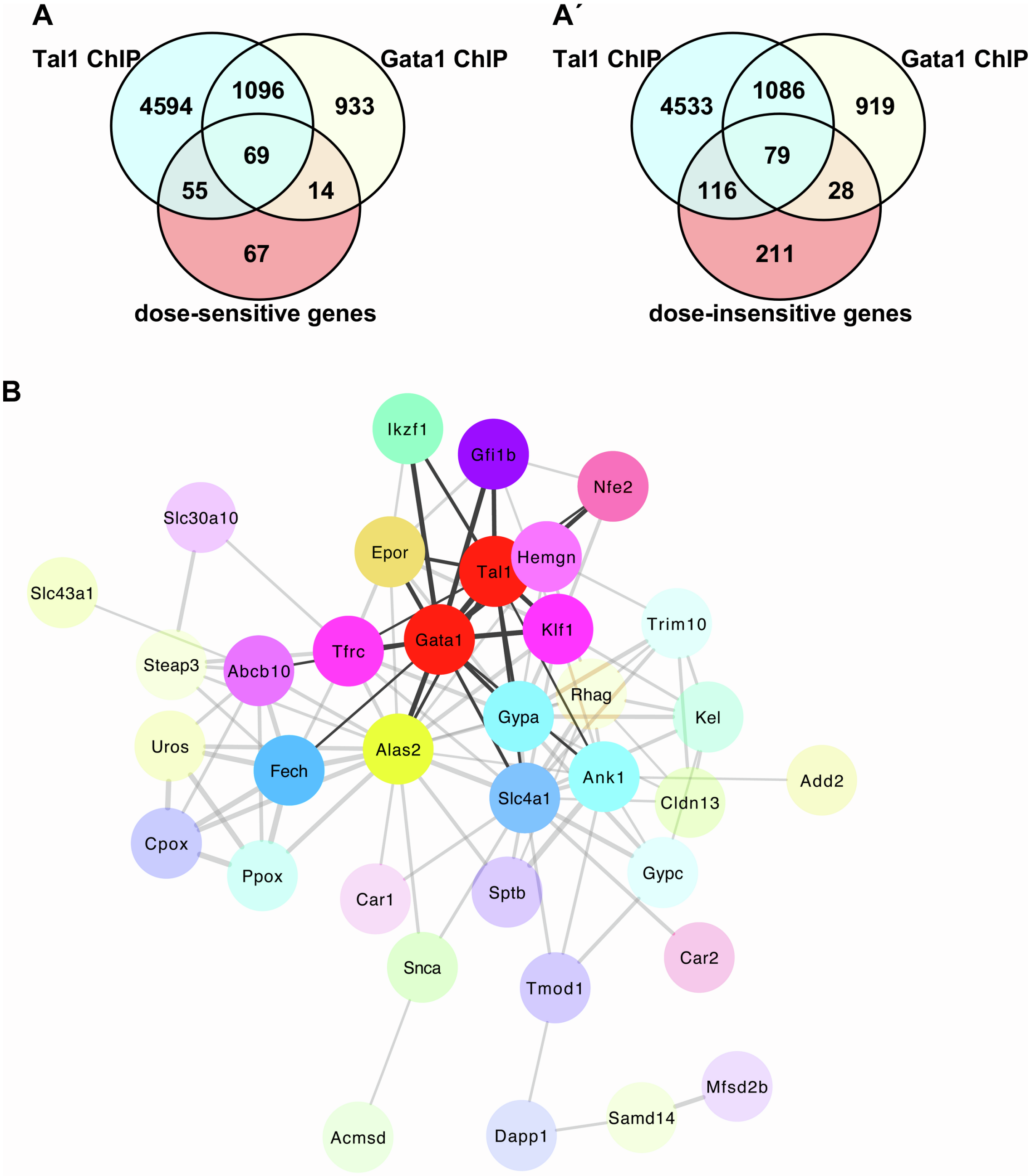
A
Tal1 ChIP
Gata1 ChIP
4594
1096
933
69
55
14
67
dose-sensitive genes
A´
Tal1 ChIP
Gata1 ChIP
4533
1086
919
79
116
28
211
dose-insensitive genes
B
Ikzf1
Gfi1b
Nfe2
Slc30a10
Epor
Tal1
Hemgn
Slc43a1
Trim10
Tfrc
Gata1
Klf1
Steap3
Abcb10
Rhag
Gypa
Kel
Uros
Alas2
Fech
Ank1
Add2
Slc4a1
Cldn13
Cpox
Ppox
Car1
Sptb
Gypc
Snca
Tmod1
Car2
Mfsd2b
Samd14
Acmsd
Dapp1

Figure S5. Etv2 dose-sensitive genes are directly co-regulated by Tal1 and Gata1. Related to Figure 6. Venn diagrams of the intersection of Etv2 dose-sensitive differentially expressed genes (DEGs) (A) and dose-insensitive DEGs (A′) with Tal1 and Gata1 ChIP-seq datasets showing that 33% (69/205) of all Etv2 dose-sensitive genes are directly bound by both Tal1 and Gata1 whereas only 18% (79/444) of dose-insensitive genes are bound by Tal1 and Gata1. (B) Cytoscape network interaction visualization for dose-sensitive genes bound by Tal1 and Gata1. 42 of the 69 co-bound genes are part of a direct and indirect erythropoietic network. Gata1 and Tal1 (red circles) are the central nodes in the network with solid-colored circles and dark lines representing direct first-degree interactions with either Gata1 or Tal1.

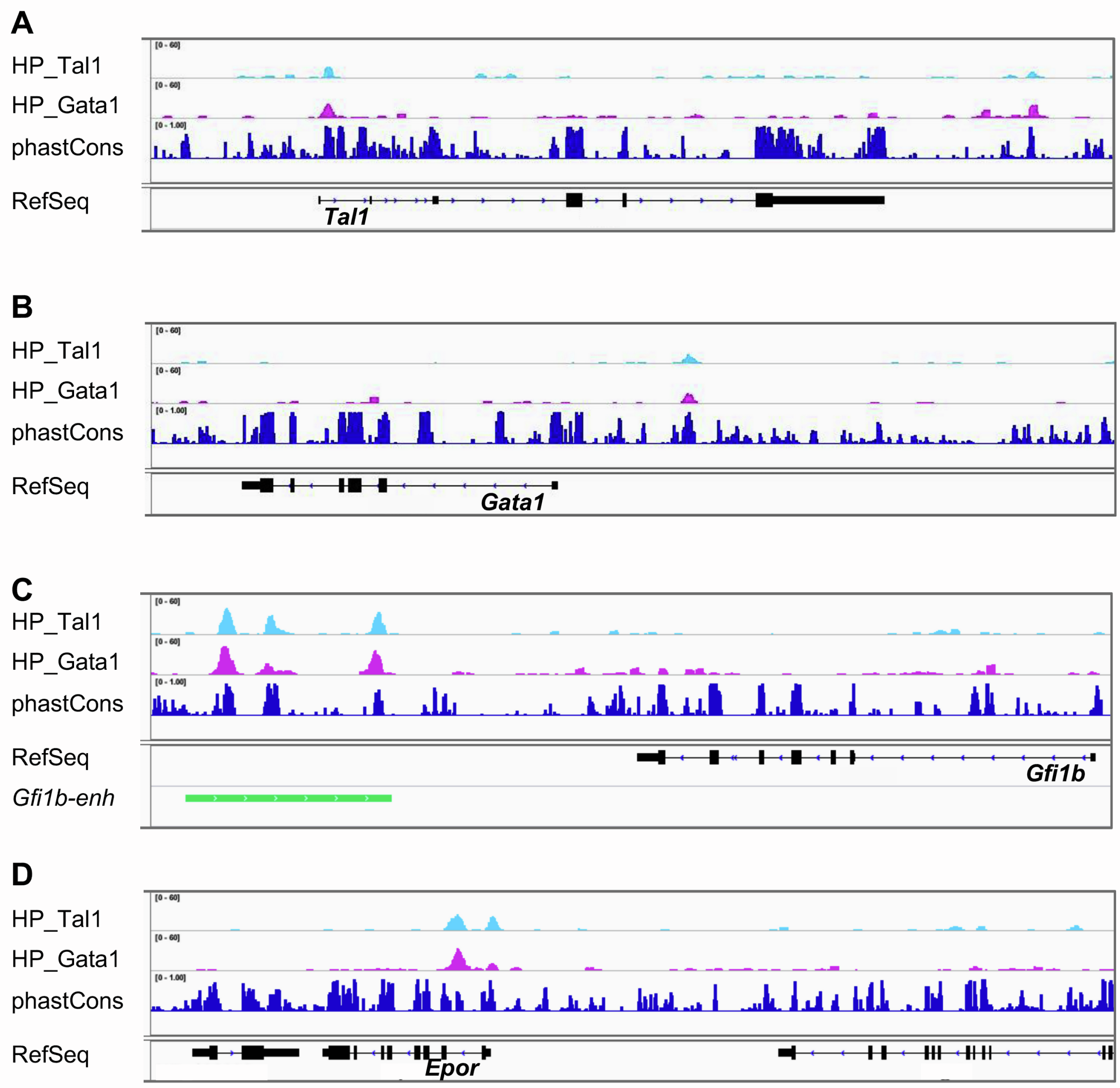
A
HP_Tal1
HP_Gata1
phastCons
RefSeq
Tal1
B
HP_Tal1
HP_Gata1
phastCons
RefSeq
Gata1
C
HP_Tal1
HP_Gata1
phastCons
RefSeq
Gfi1b
Gfi1b-enh
D
HP_Tal1
HP_Gata1
phastCons
RefSeq
Epor

Figure S6. Co-localization of Tal1 and Gata1 binding sites near indirect Etv2-dependent hematopoietic genes. Related to Figure 6. Browser tracks of ChIP-seq data showing co-occupancy of Tal1 (blue peaks) and Gata1 (purple peaks) transcription factors at and around examples of Etv2 dose-sensitive gene loci, including *Tal1* (A), *Gata1* (B), *Gfi1b* (C), and *Epor* (D) in hematopoietic progenitor cells. The phastCons track indicates sequence conservation at each of these regions across 60 vertebrate species. The green bar for *Gfi1b* (C, *Gfi1b-enh*) indicates the genomic location of the *Gfi1b* enhancer tested for *Etv2* dose-sensitivity.

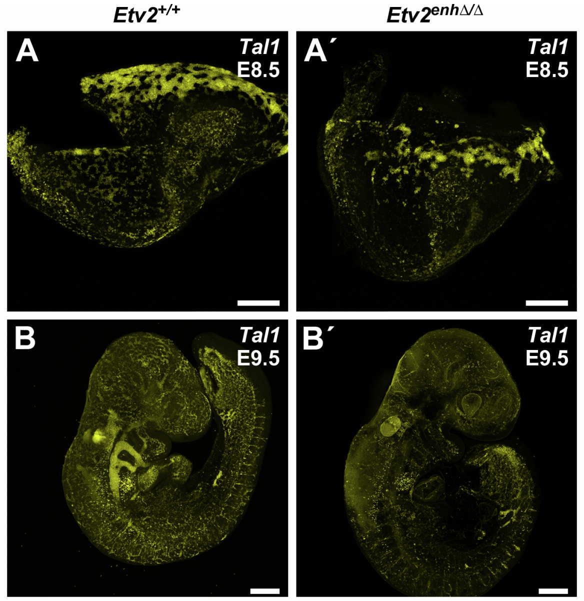

Etv2+/+
Etv2enhΔ/Δ
A
Tal1
E8.5
A´
Tal1
E8.5
B
Tal1
E9.5
B´
Tal1
E9.5

Figure S7. *Tal1* expression is reduced throughout *Etv2*$^{enh\Delta/\Delta}$ hypomorphic mutant embryos. Related to Figure 7. *In situ* hybridization analysis of *Tal1* by RNAscope shows globally reduced *Tal1* mRNA expression in *Etv2*$^{enh\Delta/\Delta}$ mutant embryos (A′,B′) compared to wild type embryos (A,B) at E8.5 (A,A′) and E9.5 (B,B′). Scale bars, 500μM. For A,B, n=3 biological replicates/genotype.
