# Supplementary material for: Differential Etv2 threshold requirement for endothelial and erythropoietic development
Source: Cell Rep. Author manuscript; Available in PMC 2022 Jun 16. (PMC9203129; doi:10.1016/j.celrep.2022.110881)
Supplement: 5 [file NIHMS1812256-supplement-5.docx]

**Table S4. Oligonucleotide sequences used for genotyping and qPCR.**

| ***Gene*** | **Primer name** | **Primer sequence (5'->3')** | **Product size** |
| --- | --- | --- | --- |
| ***Etv2 Δ*** | Etv2-I3F | cacactagagctgagttcagacc | I3F+I5R: 1401 bp (WT) |
|  | Etv2-I5R | ggaccagggtcttagctgc | I3F+I5R: 240bp (Δ) |
|  | Etv2-E4R3 | atgtggctctatcagactgcttg | I3F+E4R3: 618 bp (WT) |
| ***Etv2 enhΔ*** | LBL-F | GCCTCTTCTCCCACCACATA | LBL F+R = 2157 bp (WT) |
|  | LBL-R | TTGGCACTAGAATGCACAGC | LBL F+R = 447 bp (enhΔ) |
|  | EED-WTF2 | GTGGAACCCTTCCCAGTTC | EEDWTF2+LBL-R = 650 bp (WT) |
| ***LacZ*** | LacZ_F | tttaacgccgtgcgctgttcg | 275bp |
|  | LacZ_R | atccagcgatacagcgcgtcg |  |
| ***Etv2*** | qEtv2-F | CAGAGTCCAGCATTCACCAC | 113 bp |
|  | qEtv2-R | AGGAATTGCCACAGCTGAAT |  |
| ***Cdh5*** | qCdh5-F | TCCTCTGCATCCTCACCATC | 122 bp |
|  | qCdh5-R | GTAAGTGACCAACTGCTCGT |  |
| ***Gata1*** | qGata1-F | TGCAATGCCTGCGGCCTCTA | 167 bp |
|  | qGata1-R | AAGCCACCAGCTGGTCCTTC |  |
| ***ActB*** | qActB-F | AGTGTGACGTTGACATCCGT | 120 bp |
|  | qActB-R | TGCTAGGAGCCAGAGCAGTA |  |
| ***EfnB2*** | qEfnb2 - F | CGAGGTGGCAACAACAATGG | 105 bp |
|  | qEfnb2 - R | ATAGTCCCCGCTGACCTTCT |  |
| ***Pecam1*** | qPecam-F | CCAAAGCCAGTAGCATCATGGTC | 144 bp |
|  | qPecam-R | GGATGGTGAAGTTGGCTACAGG |  |
| ***Hbb-Ƴ*** | qHbb-y - F | CAAGCTACATGTGGATCCTGAGAA | 77 bp |
|  | qHbb-y-R | TGCCGAAGTGACTAGCCAAA |  |
| ***Hbb-bh1*** | qHbb-bh1 - F | AGGCAGCTATCACAAGCATCTG | 112 bp |
|  | qHbb-bh1 - R | AACTTGTCAAAGAATCTCTGAGTCCAT |  |
| ***GypA*** | qGypa-F | ACTCCTGTGGTGGCTTCAACTG | 141 bp |
|  | qGypa-R | GTGTGGTGAGACAGGCTGTTCT |  |
| ***Gfi1b*** | qGfi1b-F | GGAGATGTTGCTGAACCAGAGC | 143 bp |
|  | qGfi1b-R | CCAAGGTATCCCAGGAGAAGCT |  |
| ***Nr2f2*** | qNr2f2 - F | CGCCGAGTATAGCTGCCTCAAG | 130 bp |
|  | qNr2f2 - R | CTGGCTCCTAACGTACTCTTCC |  |
| ***Scl/Tal1*** | qTal1-F | GCCAGCCGCTCGCCTCACTA | 143 bp |
|  | qTal1-R | CCGCACTACTTTGGTGTGAGGA |  |
